# TDRPFormer: Texture-Debiased Reliable Prototype Transformer for Pixel-Level Steel Surface Defect Segmentation

**DOI:** 10.3390/s26175602

**Published:** 2026-09-03

**Authors:** Mingxiang Zhu, Zhizhen Li, Hongyan Sun, Yu Wang, Jue Wang

**Affiliations:** School of Electric Power Engineering, Nanjing Normal University Taizhou College, Taizhou 225300, China; 20101010@nnutc.edu.cn (M.Z.); 20051036@nnutc.edu.cn (Z.L.); 20201020@nnutc.edu.cn (Y.W.); 20121006@nnutc.edu.cn (J.W.)

**Keywords:** steel surface defect segmentation, industrial visual inspection, Transformer, texture debiasing, reliable prototype routing, structural continuity modulation

## Abstract

Steel surface defect segmentation is a critical task in industrial visual inspection, with direct implications for quality assessment, process control, and in-service safety. However, complex steel-surface images often contain strong background textures, local reflections, machining traces, and low-contrast defects, which cause existing methods to be distracted by normal textures and to produce false positives or missed detections in slender structures, blurred boundaries, and weak-response regions. To address these challenges, we propose a Texture-Debiased Reliable Prototype Transformer (TDRPFormer) for pixel-level steel surface defect segmentation. Within a query-driven mask prediction framework, TDRPFormer first derives pixel-level confidence and boundary cues from intermediate segmentation priors and then aggregates normal texture prototypes from high-confidence background regions. A Texture Debiasing Module (TDM) is introduced to suppress the interference of normal textures in defect representations. Next, a Reliable Prototype Routing (RPR) module compresses the debiased spatial features into a small set of high-reliability prototypes, thereby reducing ineffective interactions between queries and redundant background pixels. Finally, a Structural Continuity Modulation (SCM) module enhances high-resolution mask features, improving the recovery of slender defects, weak-response regions, and blurred boundaries. We conduct systematic experiments on the ESDIs-SOD and NEU-DET steel surface defect datasets. TDRPFormer achieves the best overall performance on both datasets. On ESDIs-SOD, the mean absolute error (MAE), weighted F-measure ($F_{\beta }^{w}$), S-measure ($S_{\alpha }$), and mean E-measure ($mE_{\xi }$) reach 0.0185, 0.8824, 0.9070, and 0.9626, respectively. On NEU-DET, the corresponding values are 0.0210, 0.8858, 0.9017, and 0.9662. Further ablation studies and response visualizations confirm the complementary roles of texture debiasing, reliable prototype routing, and structural continuity modulation. These results demonstrate that TDRPFormer improves discriminability, stability, and structural recovery under complex industrial backgrounds, providing an effective solution for pixel-level steel surface defect segmentation.

## 1. Introduction

Steel is widely used in critical fields such as rail transit, bridge engineering, energy equipment, and advanced manufacturing, where surface quality is directly associated with component reliability, downstream processing stability, and final product safety. Therefore, steel surface defect detection has long been regarded as an important research topic in industrial visual inspection. In recent years, substantial progress has been achieved in steel surface defect analysis in terms of detection accuracy, robustness, and automation, owing to the rapid development of deep learning. Accordingly, related studies have gradually shifted from handcrafted features and rule-based discrimination methods to data-driven methods based on convolutional neural networks, attention mechanisms, and Transformers [1,2,3,4,5,6]. Meanwhile, the increasing demand for defect area, boundary morphology, and spatial distribution information in industrial quality analysis has made pixel-level defect segmentation more valuable than defect detection methods that only output bounding boxes [7,8,9].

**Figure 1:**
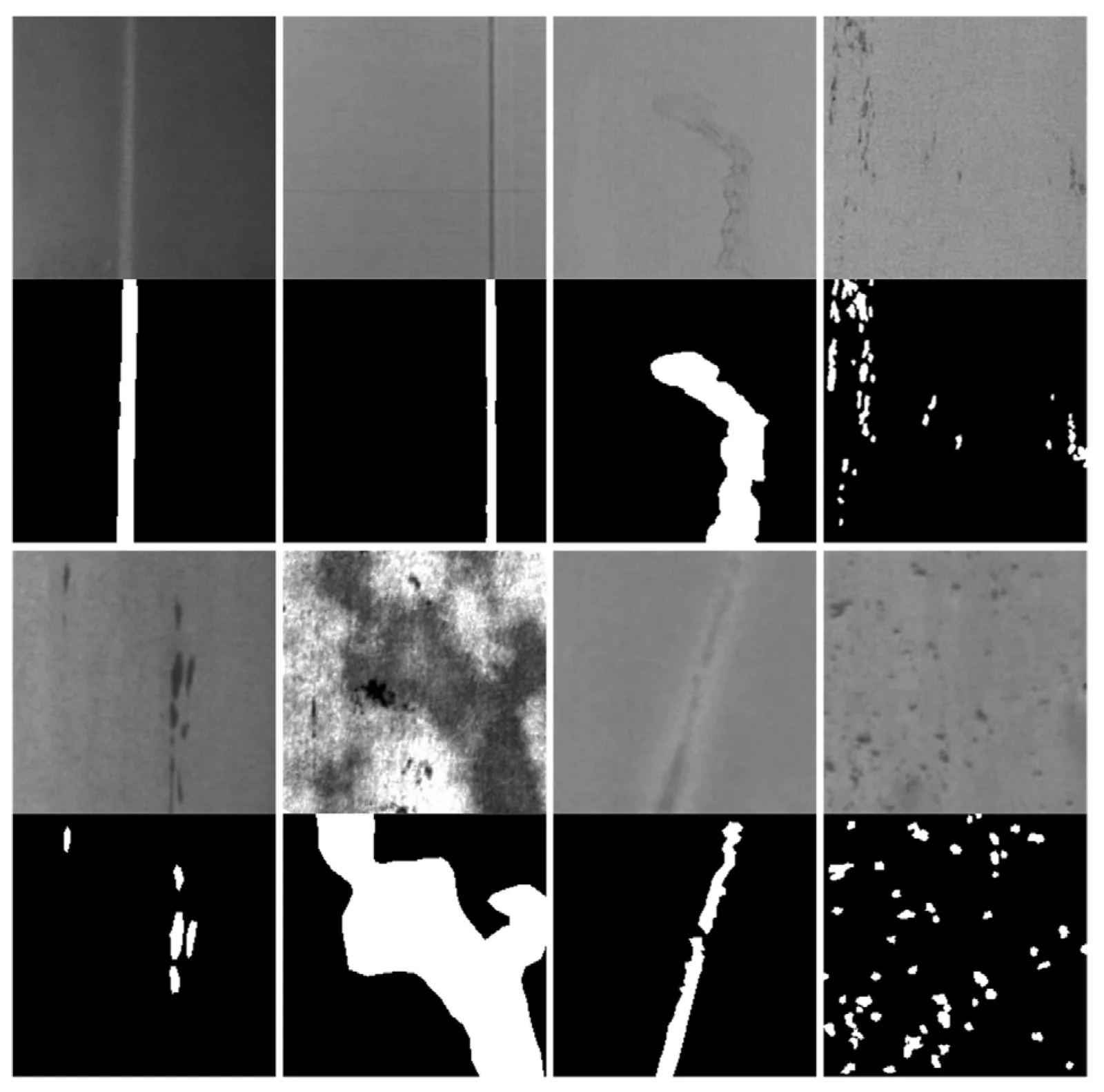
Representative steel surface defect examples and the corresponding segmentation challenges. From top to bottom and from left to right, the examples are scratches, welding line, water spot, foreign matter inclusion, patches, roll printing, and oxide scale of temperature system.

Although existing studies have substantially advanced steel surface defect analysis, pixel-level defect segmentation in complex industrial scenarios remains challenging. Steel surface images are often affected by rolling textures, reflective patterns, processing traces, and local noise. These normal background structures often show high local similarity to true defects, which may lead to false detections [1,3,6,10,11,12]. Representative examples are shown in Figure 1. In addition, defects such as cracks, scratches, inclusions, and pinholes are usually characterized by elongated shapes, low contrast, large-scale variations, and blurred boundaries. These characteristics make it difficult for segmentation models to preserve both regional completeness and boundary accuracy [10,11]. Therefore, the stable suppression of normal textures and the accurate recovery of true defect regions under complex background interference remain key issues in pixel-level steel surface defect segmentation.

From the perspective of recent research trends, steel surface defect analysis has gradually evolved from bounding-box-level detection to pixel-level segmentation, lightweight modeling, and global context-aware architecture design [12,13,14]. Strategies such as multi-scale feature fusion, contextual aggregation, attention modeling, and lightweight Transformers have been shown to enhance the representation of complex defect patterns. As a result, steel surface defect analysis has been promoted from coarse-grained recognition toward fine-grained understanding. Meanwhile, rapid advances in generic visual segmentation have provided new insights for industrial defect segmentation. Strong transferability in open scenarios has been demonstrated by foundation segmentation models such as Segment Anything [15]. The unity and effectiveness of query-based mask prediction paradigms in dense prediction tasks have been further validated by Mask2Former and dynamic query-based methods [16,17]. Inspired by these advances, foundation model adaptation, query interaction, and prototype modeling have also been explored for surface defect analysis in industrial vision [13,14]. However, steel surface defect segmentation differs substantially from natural image segmentation and general industrial anomaly detection. Normal textures in steel surface images usually exhibit strong repetitiveness, local similarity, and high-frequency responses, which may cause background structures to dominate the feature space. Although positive progress has been achieved, most existing methods focus mainly on enhancing defect responses, while the distribution of normal background textures is rarely modeled explicitly [6,14]. In this case, false detections are not always caused by insufficient defect responses but are often induced by appearance confusion between normal textures and true defects. Therefore, background texture interference in defect discrimination cannot be effectively reduced by relying only on attention enhancement or feature aggregation.

Moreover, spatial reliability in complex industrial images is usually uneven. Regions with blurred boundaries, weak responses, and texture interference usually contain higher uncertainty. Without reliability constraints, query updating and mask prediction may be continuously disturbed by low-quality background features [18,19]. Meanwhile, the recovery of elongated cracks and discontinuous edges depends not only on region-level semantic discrimination but also on the effective preservation of structural continuity. Related crack segmentation studies have shown that structural continuity has an important influence on the segmentation quality of elongated and low-contrast targets [20]. In recent years, advances in uncertainty modeling, frequency feature analysis, and prototype learning have provided useful insights for addressing these issues. It has been shown that uncertainty estimation can help identify low-reliability regions and improve the robustness of feature learning [21]. Frequency feature correction has been found to improve the preservation of boundary and detail information [22]. Prototype learning and prototype matching can be used to construct compact and structured discriminative representations in dense prediction tasks, thereby reducing redundant background interference [23,24]. However, most of these methods focus on only one aspect, such as reliability estimation, detail recovery, or regional representation. A unified modeling framework has not yet been established for the combined challenges of strong normal texture interference, weak defect responses, and insufficient boundary continuity in steel surface defect segmentation.

Based on the above analysis, a steel surface defect segmentation framework based on texture debiasing and reliable prototype routing, named Texture-Debiased Reliable Prototype Transformer (TDRPFormer), is proposed in this study. Rather than only enhancing defect responses, TDRPFormer explicitly reduces normal texture interference and refines defect structures through prediction-conditioned feature processing. Stage-wise predictions are used to suppress background-dominant texture components, guide reliability-aware prototype routing, and provide structural cues for high-resolution mask correction. The detailed design of the three modules is described in Section 3.

The main contributions of this study are summarized as follows:

(1) A query-driven framework, TDRPFormer, is proposed for pixel-level steel surface defect segmentation. The framework jointly addresses normal-texture interference, unreliable spatial interaction, and structural discontinuity under complex industrial backgrounds;
(2) A texture-debiasing mechanism is designed. Normal texture prototypes are explicitly modeled from high-confidence background regions, and current-scale features are adaptively debiased, thereby alleviating the visual confusion between normal textures and real defects and reducing the risk of false positives caused by complex backgrounds;
(3) Reliable prototype routing and structural continuity modulation strategies are developed. The former guides query updating toward highly reliable defect cues while reducing interference from redundant background features. The latter strengthens the structural recovery of slender, weak-response, and boundary-blurred defects, thereby improving pixel-level segmentation performance in complex industrial scenarios.

## 2. Related Work

### 2.1. Steel Surface Defect Detection and Pixel-Level Segmentation

Steel surface defect analysis is a representative task in industrial visual inspection. Research in this area has gradually evolved from early handcrafted features, texture descriptors, and rule-based discrimination to automated detection and segmentation frameworks centered on deep learning. In recent years, improvements in steel surface defect recognition have mainly been pursued through feature alignment, lightweight detection, multi-scale fusion, attention enhancement, and global context modeling. For example, hierarchical training and feature alignment mechanisms have been used to improve feature consistency in steel surface defect recognition [4]. An improved YOLO framework was developed by incorporating the Swin Transformer and adaptive feature fusion, and enhanced detection performance on hot-rolled steel strip defects was reported [5]. Global attention, cascaded fusion, and context-guided refinement strategies have also been introduced to strengthen defect representation under complex industrial backgrounds [6,25]. Although these methods have substantially improved box-level localization and recognition accuracy, their outputs are still mainly intended for defect localization and cannot directly satisfy industrial requirements for defect area, boundary morphology, and spatial distribution.

For this reason, research on steel surface defects has gradually expanded from object detection to pixel-level segmentation. Existing segmentation methods have mainly been developed around encoder–decoder architectures, pixel-wise interaction modeling, and Transformer-based feature fusion. For instance, Resformer–Unet combined ResNet with a Transformer to improve local detail representation and global semantic modeling in strip-steel defect segmentation [26]. Pyramid Cross Attention Network enhanced interactions among pixel regions at different scales through pyramid cross-attention [8]. GDALR integrated global dual attention with local representation to improve defect-region modeling under complex backgrounds [9]. Recent studies have also improved surface defect segmentation from the perspectives of prediction localization and pixel-level confidence estimation. For example, the Localization and Pixel-Confidence Network alleviates region imbalance and mis-segmentation of internal defect gaps through defect localization and pixel-confidence refinement [27]. Other studies have exploited deep change features between defective and defect-free images to achieve pixel-level defect segmentation under complex backgrounds [28]. These studies indicate that multi-scale context modeling, local detail recovery, and prediction refinement are important for defect segmentation. However, most existing methods are still mainly developed around defect response enhancement or feature fusion.

When pixel-level annotation is costly and industrial scenarios vary substantially, low-annotation and cross-scenario segmentation strategies have also been widely investigated. Hybrid supervised learning can reduce the dependence of surface defect detection on fine-grained pixel annotations [29], while joint weakly and fully supervised learning can improve the use of limited annotation information [30]. Meanwhile, memory attention, multi-reasoning mechanisms, and few-shot semantic segmentation methods have been used to enhance the representation of complex defect patterns and alleviate defect sample scarcity [31,32]. For cross-scenario applications, uncertainty-guided domain adaptation has further been adopted to reduce distribution shifts across different industrial environments [33].

In recent few-shot industrial defect segmentation studies, multi-scale adaptive prototype Transformers [7], background-weakening generalization networks [34], and segmentation methods that combine prior knowledge, background removal, and texture enhancement [35] have been proposed to improve model adaptability to unseen defect categories and complex backgrounds under limited annotation conditions. It should be noted that these methods mainly focus on sample scarcity, support-query transfer, or cross-scenario generalization. In contrast, this study adopts a fully supervised pixel-level segmentation setting and focuses on false responses caused by the local similarity between normal textures and true defects within the same steel surface image. This problem is addressed through explicit texture debiasing and reliability-conditioned feature modeling.

The development of visual foundation models has provided a new transfer pathway for industrial defect segmentation. Existing studies have shown that generic segmentation foundation models may be limited by weak defect appearances, complex background textures, and domain-specific feature distribution shifts when directly applied to industrial scenarios. To address this issue, DefectSAM applies SAM to pixel-level surface defect detection through hierarchical feature adaptation [13]. Visual foundation model methods for few-shot industrial defect segmentation enhance domain adaptability by using reference defect prompts and metric learning [21]. PA-SAM further transforms SAM into an end-to-end industrial defect segmentation framework by integrating parameter-efficient fine-tuning, multi-scale partial convolution aggregation, and automatic prompt generation [36]. These methods mainly focus on reducing the domain gap between the pretrained knowledge of foundation models and target industrial scenarios, or on lowering the cost of manual prompts and annotations. In contrast, this study does not focus on foundation model transfer but, instead, addresses pixel-level false responses caused by the high similarity between normal textures and true defects within the current image.

Texture-aware modeling and background interference suppression are also important directions in industrial defect segmentation. Existing studies have reduced the influence of complex textures on defect recognition through low-level texture enhancement, background removal, frequency–domain feature modeling, and anomaly detection strategies [37,38]. These studies suggest that rolling textures, reflective stripes, and processing traces on manufactured surfaces are not irrelevant background patterns but may produce local responses similar to those of true defects. However, most existing methods address specific tasks such as few-shot generalization, anomaly detection, or frequency-domain detail enhancement. Relatively few studies on surface defect segmentation explicitly represent normal texture as a suppressible, image-adaptive background reference and further integrate this with query updating and high-resolution structural recovery. Against this backdrop, this paper focuses on the synergistic relationship among normal texture debiasing, reliable region aggregation, and the restoration of structural continuity.

### 2.2. Query-Driven Segmentation and Reliable Structural Modeling

In recent years, general visual segmentation has gradually shifted from traditional pixel-wise classification frameworks to query-centered mask prediction paradigms. Segment Anything demonstrated the transfer potential of foundation segmentation models in open scenarios [15]. Mask2Former unified semantic, instance, and panoptic segmentation through masked attention [16]. Dynamic focus-aware positional queries further showed that query initialization and updating strategies are important for localizing complex regions and generating accurate masks [39]. Compared with conventional pixel-classification methods, query-driven segmentation can form more compact region-level representations through global interaction and is, therefore, better suited to targets with irregular shapes, large-scale variation, and complex backgrounds. In industrial defect segmentation, WPFormer further applies wavelet-enhanced cross-attention and prototype-enhanced cross-attention to query updating [14]. This indicates that query-driven mechanisms can provide a representation strategy for complex defect regions that differs from conventional pixel classification. However, this type of prototype interaction is mainly used for feature representation enhancement. In contrast, this study does not use prototype interaction to replace complete spatial feature interaction. Instead, after global contextual interaction with the debiased spatial features, reliability cues provided by current predictions are used to constrain the prototypes, which then serve as complementary refinement information for the queries.

Prototype representation learning provides another important complement to query-driven segmentation. The core idea of prototype learning is to extract a small number of representative structured vectors from large spatial feature sets, thereby compressing redundant pixel information and strengthening region-level semantic representation. Prototype-Based Semantic Segmentation systematically discussed the role of prototypes in connecting pixel-level features with region-level semantics [40]. Prototype–anchor contrast enhanced local discriminative relations through prototype–anchor comparison [41]. Query-guided prototype evolution networks improved the robustness of few-shot segmentation by dynamically updating prototypes [42]. Methods that directly use prototypes as queries further suggest an intrinsic consistency between prototypes and queries [43]. Multi-granularity logical prototypes have also shown that structured prototypes can improve category discriminability and regional consistency in dense prediction [44]. Collectively, these studies indicate that both queries and prototypes can serve as effective tools for compressing spatial features and organizing region-level semantics.

However, most existing query-driven and prototype-learning methods are designed for natural images, general semantic segmentation, or few-shot scenarios, where prototypes are usually aggregated from raw spatial features or support-set features. For steel surface defect segmentation, relying only on region-level semantic aggregation may still cause queries to be disturbed by redundant background responses. This problem becomes particularly pronounced when rolling textures, reflective stripes, and low-contrast defects coexist. In this case, query updating requires not only region-level semantic information but also reliability constraints provided by current predictions. Therefore, prediction confidence and boundary responses can be used as auxiliary conditions for reliable prototype aggregation. In this way, prototype information provides complementary refinement for the queries after full spatial-context interaction.

Beyond region representation, prediction reliability and boundary structure recovery also directly affect pixel-level segmentation quality in complex industrial images. Studies on anomaly detection in complex industrial imagery have shown that anomalous regions are often intertwined with structured backgrounds, which challenges model discriminability [45]. Crack segmentation studies have likewise indicated that blurred non-crack boundary regions can interfere with the recognition of true crack structures [46]. For this reason, uncertainty modeling and reliability estimation have gradually been introduced to improve segmentation stability. An uncertainty-aware dynamic mixed pseudo-labeling method improved semi-supervised defect segmentation by modeling pseudo-label reliability [47]. Uncertainty quantification in image-level crack segmentation further showed that reliability analysis is important in blurred-boundary and weak-response regions [48]. It should be noted that the confidence maps used in this study are deterministic reliability cues constructed from stage-wise predictions. They are used for feature modulation and prototype routing, rather than as calibrated uncertainty estimates. Their consistency with pixel-level prediction errors and their role in constructing TDM background weights are quantitatively examined in Section 4.3.7.

Structural continuity and boundary preservation are also key issues in fine-grained defect segmentation. Cracks, scratches, and strip-like defects are typically slender, discontinuous, and low in contrast. If only region-level semantic aggregation is relied upon, boundary fracture and local omission can easily occur. To address this issue, non-crack boundary region modeling has been used to improve the discrimination between cracks and background [46]. Topology-aware Mamba architectures have been introduced to strengthen topological continuity modeling in crack segmentation [49]. Lightweight structure-aware Mamba further improved the recovery of slender crack structures [20]. Multidimensional structural information fusion networks also verified the effectiveness of structural priors for restoring crack boundaries in concrete imagery [50]. In addition, frequency-based feature correction has been used to improve boundary-detail preservation in dense prediction tasks [22]. However, in steel surface defect images, high-frequency responses do not necessarily correspond to real defects. Normal rolling textures, reflective streaks, and machining traces may also generate strong local responses. As a result, indiscriminate enhancement of boundaries or high-frequency details may further amplify background interference. A more appropriate strategy is to selectively preserve and exploit defect boundaries and local structures under reliability guidance.

Despite this progress, explicit modeling of normal texture interference remains insufficient in complex steel surface segmentation, where repetitive background patterns can produce defect-like responses. This limitation motivates the texture-debiased modeling adopted in this study.

## 3. Methodology

### 3.1. Overall Network Architecture

**Figure 2:**
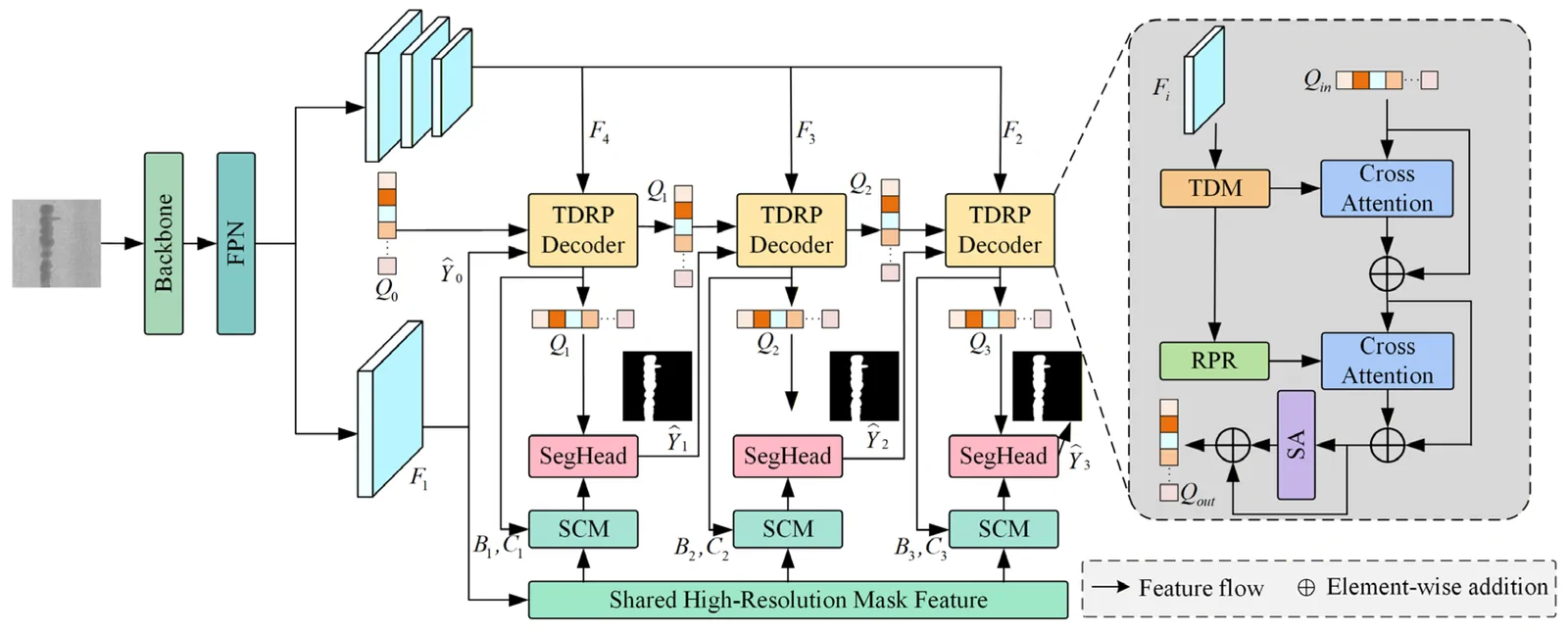
Overall architecture of the proposed TDRPFormer. The network consists of multi-scale feature encoding, three-stage query refinement, and a shared high-resolution mask prediction branch. Specifically, $F_{4}$, $F_{3}$, and $F_{2}$ are used sequentially to update the query representations, whereas the high-resolution feature $F_{1}$ is used to construct the shared mask feature branch for stage-wise defect prediction. The final output is obtained by fusing predictions from multiple decoding stages.

The overall architecture of the proposed TDRPFormer is illustrated in Figure 2. The network follows a query-driven encoder–decoder design and consists of three main components: multi-scale feature encoding, query-based decoding blocks, and a structurally enhanced mask prediction head.

Given an input steel surface image $I\in R^{3\times H\times W}$, PVTv2 [51] is first adopted as the backbone to extract four-stage features at resolutions of 1/4, 1/8, 1/16, and 1/32. These features are then projected to 64 channels by 1 × 1 convolutions and fed into a vanilla feature pyramid network (FPN) [52], yielding multi-scale features $F_{1},F_{2},F_{3},F_{4}$. Among them, the high-resolution feature $F_{1}$ is used to construct a shared high-resolution mask feature branch, whereas $\left\{F_{2},F_{3},F_{4}\right\}$ serve as multi-scale semantic features for stage-wise query updating.

To achieve region-level feature aggregation, a set of N = 16 learnable query embeddings with channel dimension C = 64 is adopted. For query initialization, the shared high-resolution mask feature is first projected to 64 channels and used as the memory of a two-layer Transformer decoder. A sine positional encoding is added to the memory tokens, whereas the learnable query embeddings are used as query positional embeddings. The query content is initialized as an all-zero tensor. The decoder output is then used as the initial query representation for subsequent stage-wise refinement. The query initializer uses eight attention heads, a feed-forward dimension of 2048, ReLU activation, and dropout of 0.1.

Unlike architectures in which the final mask is generated only at the last decoding stage, prediction is performed at the initial stage and after each of the three refinement stages using the shared high-resolution mask feature branch. During training, the initial prediction and the three stage-wise predictions are supervised using the same binary cross-entropy and IoU losses. At inference, the initial prediction is used only to provide the coarse prior for the first refinement stage and is not included in the final fusion. Let ${\hat{Y}}_{1}$, ${\hat{Y}}_{2}$, and ${\hat{Y}}_{3}$ denote the logits predicted after the three refinement stages. The final logit map is computed by element-wise summation, ${\hat{Y}}_{final}={\hat{Y}}_{1}+{\hat{Y}}_{2}+{\hat{Y}}_{3}$, and is then bilinearly upsampled to the input resolution. The final probability map is obtained by applying the Sigmoid function to ${\hat{Y}}_{final}$.

### 3.2. TDM

**Figure 3:**
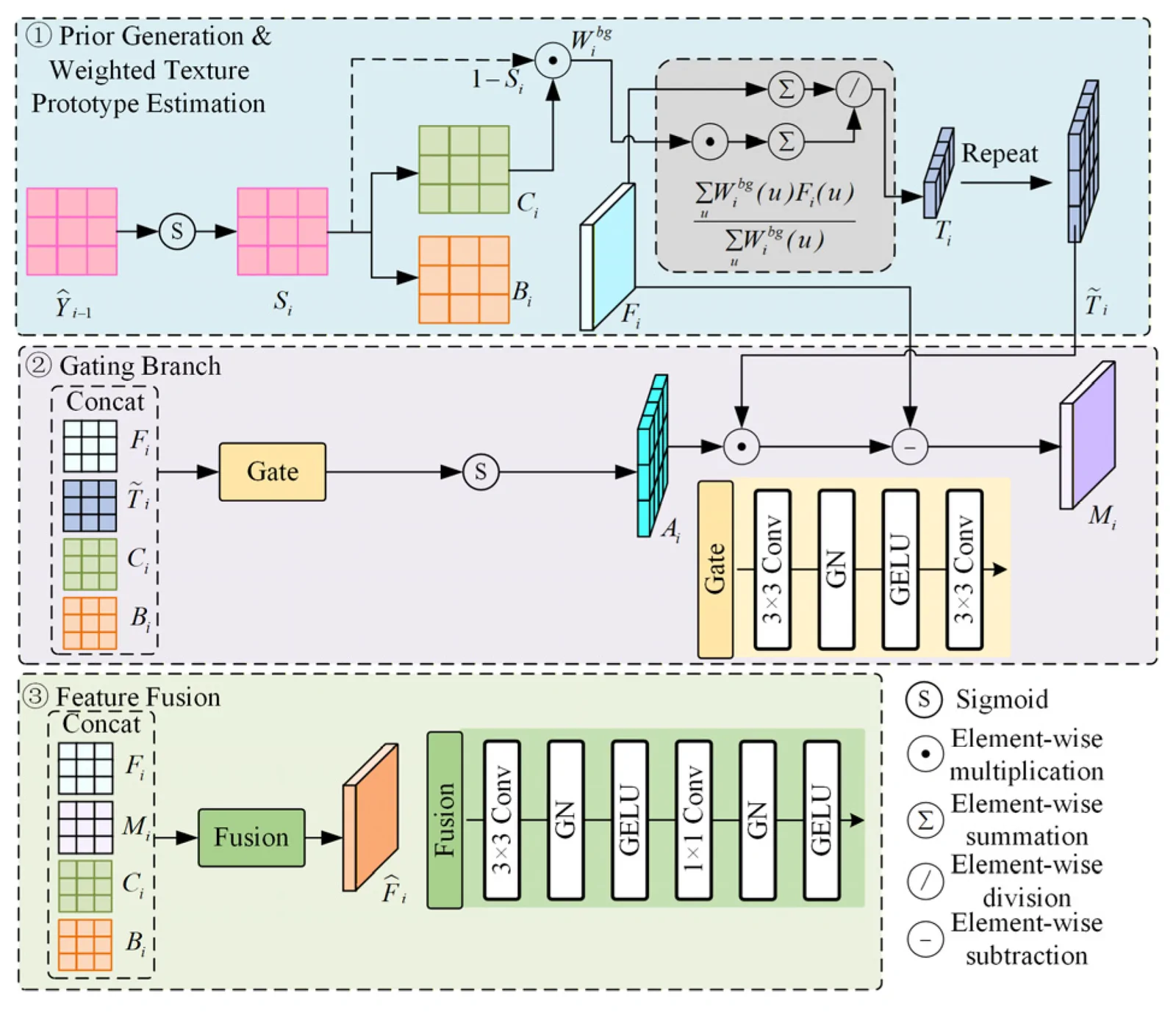
Illustration of TDM. Given the current-stage feature and its coarse segmentation prior, TDM first estimates confidence and boundary response information, and then constructs a background-guided texture prototype from highly reliable normal regions. On this basis, stage features are adaptively debiased through a gating mechanism, so that normal texture interference is suppressed and defect-related anomalous structures are highlighted.

To mitigate the interference of normal textures in defect representation, TDM is introduced. This module uses the coarse segmentation prior of the current stage to estimate background-dominated normal texture prototypes and adaptively debias stage features, thereby highlighting anomalous structures that deviate from the normal texture distribution. The structure is shown in Figure 3.

At the i-th stage, the prior used by TDM is derived from the prediction of the previous stage:

(1) $$S_{i}=\sigma ({\hat{Y}}_{i-1})$$

where σ(⋅) denotes the Sigmoid function. To characterize the reliability of predictions at different spatial locations, a pixel-wise uncertainty map is constructed based on predictive entropy:

(2) $$U_{i}=-S_{i}logS_{i}-(1-S_{i})log(1-S_{i})$$

This map is further normalized into a confidence map:

(3) $$C_{i}=1-\frac{U_{i}}{\log2}$$

where $C_{i}\in (0,1)$, and a larger value indicates a more reliable prediction at that location. The rationale behind this design is that, when the prediction probability is close to zero or one, the prediction entropy is low, indicating that the class classification at that location is relatively stable. When the prediction probability is close to the decision boundary, the prediction entropy is high, indicating that the location is more susceptible to texture interference, weak defect responses or boundary ambiguity. Consequently, the confidence map is used as a reliability metric for subsequent background modeling and feature modulation, rather than as an additional prediction output.

Meanwhile, to emphasize potential defect boundary structures, a boundary response map is constructed from the coarse segmentation prior:

(4) $$B_{i}=|\partial_{x}S_{i}|+|\partial_{y}S_{i}|$$

where $\partial_{x}$ and $\partial_{y}$ denote first-order difference operators along the horizontal and vertical directions, respectively. Unlike confidence maps, which focus on measuring the stability of classifications, boundary response maps focus on characterizing local changes in predictions and transitions in underlying structure. Together, they provide both reliability and structural cues, enabling subsequent modules to distinguish between stable background regions, ambiguous regions, and regions that may contain the boundaries of genuine defects.

To construct a representation of normal textures in the current image, features are aggregated from high-confidence background regions in a weighted manner. The background weight is first defined as:

(5) $$W_{i}^{bg}=C_{i}⊙(1-S_{i})$$

where ⊙ denotes element-wise multiplication. The normal texture prototype is then computed as:

(6) $$T_{i}=\frac{\sum_{u}\;W_{i}^{bg}(u)F_{i}(u)}{\sum_{u}\;W_{i}^{bg}(u)}$$

where u indexes the spatial position, and $T_{i}\in R^{C\times 1\times 1}$. Rather than averaging all spatial locations directly, high-confidence background regions are identified by combining background probability and confidence scores. The reason for this is that weak defects, defect edges or blurred regions affected by texture interference may contain unstable predicted responses. If these regions are included in the background statistics, genuine defect information may be mixed with normal texture patterns, thereby undermining the reliability of the debiasing process. The normal texture prototype obtained in this way can more accurately characterize the dominant background texture distribution in the current image.

To avoid the over-suppression caused by directly subtracting the background prototype, an adaptive gating mechanism is adopted for selective attenuation. Prototype subtraction with a fixed intensity is not suitable for complex steel surfaces, as normal textures may appear similar to weak defects, elongated defects or features with blurred boundaries at the local level. If the background prototype is suppressed with the same intensity across all spatial positions and feature channels, this may simultaneously weaken the effective response to genuine defects. Therefore, this paper utilizes adaptive learning to determine the gating weights based on the current feature, normal texture prototype, predicted confidence level and boundary response, in order to determine the degree of background suppression at different positions and in different channels.

After the prototype is expanded to the spatial dimension and denoted as ${\tilde{T}}_{i}$, the gating map is defined as:

(7) $$A_{i}=\sigma \left(\phi ([F_{i},{\tilde{T}}_{i},C_{i},B_{i}])\right)$$

where $\left[\cdot \right]$ denotes channel-wise concatenation, and ϕ(⋅) denotes a convolutional mapping function. The input to ϕ(⋅) contains 2C+2 channels, comprising the C-channel stage feature, the C-channel expanded texture prototype, the one-channel confidence map, and the one-channel boundary-response map. The mapping block consists of a 3 × 3 convolution that maps 2C+2 channels to C channels, followed by batch normalization and GELU activation, and a 1 × 1 convolution that preserves the C-channel dimension. With C = 64, the corresponding channel transformations are 130 to 64 and 64 to 64. The output is further normalized by the Sigmoid function to obtain the spatial–channel gating map.

Once the gating map is obtained, the normal texture prototype is weighted by element-wise multiplication and subtracted from the original feature to produce the debiased residual feature:

(8) $$M_{i}=F_{i}-A_{i}⊙{\tilde{T}}_{i}$$

Accordingly, locations dominated by normal textures receive stronger prototype subtraction, whereas anomalous structural information is relatively preserved.

Finally, to preserve the original semantic information while incorporating debiased residual cues and reliability information, the original feature $F_{i}$, residual feature $M_{i}$, confidence map $C_{i}$, and boundary response map $B_{i}$ are concatenated to obtain the debiased stage feature:

(9) $${\hat{F}}_{i}=\psi ([F_{i},M_{i},C_{i},B_{i}])$$

where ψ(⋅) denotes a convolutional fusion operation. Its input also contains 2C+2 channels, obtained by concatenating the C-channel original feature, the C-channel debiased residual feature, the one-channel confidence map, and the one-channel boundary-response map. The fusion block consists of a 3 × 3 convolution that maps 2C+2 channels to C channels, followed by batch normalization and GELU activation, and a 1 × 1 convolution that preserves the C-channel output dimension. With C = 64, the corresponding channel transformations are 130 to 64 and 64 to 64. Therefore, the output debiased stage feature has the same channel dimension as the input stage feature.

Therefore, TDM differs from conventional attention mechanisms, in which feature responses are only reweighted, because normal texture references are explicitly constructed and their interfering components are selectively subtracted across spatial locations and channels through gated modulation.

### 3.3. RPR

**Figure 4:**
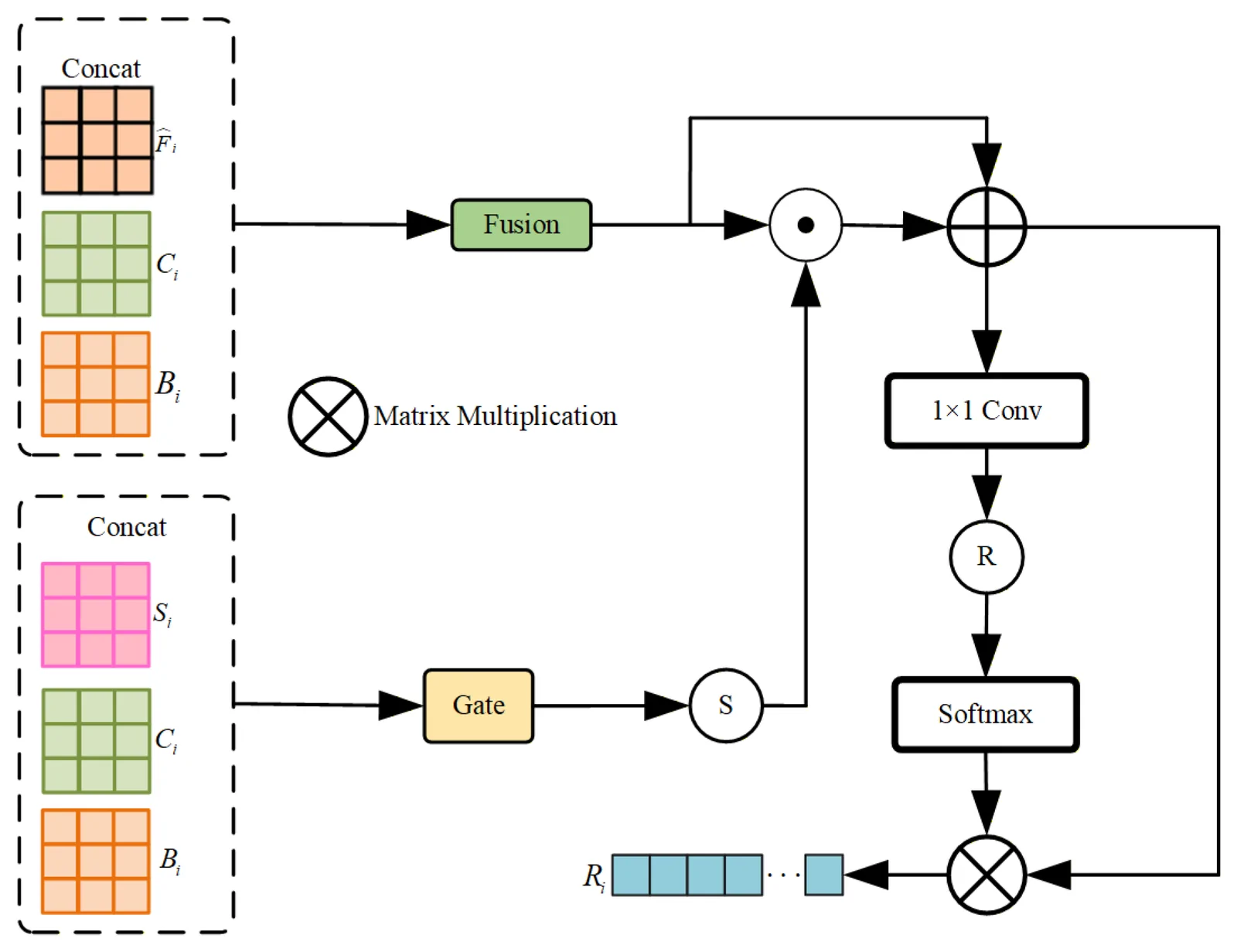
Illustration of the RPR structure. RPR takes the debiased stage features, confidence map, and boundary response map as inputs, from which reliability-conditioned routing features are first constructed. The queries first perform contextual cross-attention with the complete debiased spatial features to aggregate global semantic information. Subsequently, compact prototypes are aggregated from the routing features through spatial assignment, and a second cross-attention operation is performed between the queries and these reliability-conditioned prototypes. Through this process, prototype interaction is used as a structural complement to full spatial-context interaction rather than as its replacement.

After the debiased stage feature has been obtained, RPR is further introduced to compress and aggregate spatial information and to update query representations using highly reliable prototypes. This design reduces the interference of redundant background pixels during query updating and strengthens region-level structural representation. A similar query-guided prototype evolution strategy has previously been shown to be effective in few-shot segmentation [42]. The structure is shown in Figure 4.

Given the debiased feature ${\hat{F}}_{i}$, confidence map $C_{i}$, and boundary response map $B_{i}$, a routing feature is first constructed. Specifically, the three components are concatenated along the channel dimension and then passed through a convolutional fusion block for feature mapping:

(10) $$G_{i}=\psi ([{\hat{F}}_{i},C_{i},B_{i}])$$

Next, a routing gate map is generated from the coarse segmentation prior, confidence map, and boundary response map:

(11) $$\Gamma_{i}=\sigma (\phi (\left[S_{i},C_{i},B_{i}\right]))$$

The reliable routing feature is then defined as:

(12) $${\tilde{G}}_{i}=G_{i}⊙(1+\Gamma_{i})$$

By utilizing prediction priors, confidence scores and boundary responses in unison for the calculation of routing weights, RPR is able to reduce the influence of low-confidence background regions on prototype aggregation, whilst avoiding the excessive suppression of potential boundary regions by treating them entirely as ordinary background.

To obtain a compact region-level representation, a prototype aggregation strategy based on spatial assignment is adopted. A prototype response map is first generated using a 1 × 1 convolution:

(13) $$L_{i}=Conv_{1\times 1}\left({\tilde{G}}_{i}\right)$$

where $L_{i}\in R^{N\times H_{i}\times W_{i}}$, and N denotes the number of queries, which is kept consistent with that used in the decoder.

The spatial dimensions of $L_{i}$ are then flattened into $L_{i}^{\prime }\in R^{N\times (H_{i}W_{i})}$, and normalization is applied to obtain the prototype assignment weights:

(14) $$W_{i}^{p}=Softmax(L_{i}^{\prime })$$

where Softmax is independently computed over the spatial dimension for each prototype. The reliable routing feature ${\tilde{G}}_{i}$ is further reshaped into ${\tilde{G}}_{i}^{\prime }\in R(H_{i}W_{i})\times C$, from which the reliable prototype set is obtained as:

(15) $$R_{i}=W_{i}^{p}{\tilde{G}}_{i}^{\prime }$$

where $R_{i}\in R^{N\times C}$. This design allows different queries to correspond to different regional structural patterns.

Let the input query at the i-th stage be denoted by $Q_{i}\in R^{N\times C}$. Context interaction is first performed using the debiased stage feature. Specifically, ${\hat{F}}_{i}$ is unfolded into a sequence of spatial tokens and, together with positional encoding, is involved in multi-head cross-attention:

(16) $$Q_{i}^{c}=LN\left(Q_{i}+Attn\left(Q_{i},{\hat{F}}_{i},{\hat{F}}_{i}\right)\right)$$

where Attn(Q,K,V) denotes multi-head cross-attention, $Q_{i}$ serves as the query, the unfolded ${\hat{F}}_{i}$ serves as the key and value, and LN(⋅) denotes layer normalization.

This full-space contextual interaction ensures that queries can still access global semantic information at the current scale, rather than relying solely on a small number of compressed prototypes.

The reliable prototype set is then introduced to further update the query:

(17) $$Q_{i}^{r}=LN\left(Q_{i}^{c}+Attn(Q_{i}^{c},R_{i},R_{i})\right)$$

Finally, global dependencies among queries are further modeled through self-attention and a feed-forward network, yielding the query representation for the next stage:

(18) $$Q_{i+1}=FFN(SA(Q_{i}^{r}))$$

where SA(⋅) denotes multi-head self-attention, and FFN(⋅) denotes a feed-forward network.

It should be emphasized that prototype interaction in RPR is not designed to replace full spatial-context interaction. The queries first acquire global context from all debiased spatial features and are then further refined by reliability-conditioned prototypes, so that global semantic information and reliable local patterns can be jointly considered.

All cross-attention and self-attention operations in RPR use eight heads with a feature dimension of 64, corresponding to a per-head dimension of 8. The contextual cross-attention, prototype cross-attention, and self-attention blocks all follow a residual connection and Layer Normalization design. After query interaction, a feed-forward network is applied with the structure 64 to 256 to 64. The feed-forward network uses a ReLU activation, zero dropout, residual addition, and Layer Normalization. These settings are shared across the three stage-wise RPR blocks.

### 3.4. SCM

Cracks and scratches in steel surface defects often exhibit slender and discontinuous structural patterns. If only high-level semantic region discrimination is relied upon, boundary fracture and local omission can easily occur. To address this issue, SCM is introduced into the shared high-resolution mask branch, so that the prediction head at each stage can incorporate stage-specific reliability information to structurally enhance high-resolution mask features.

Let the shared high-resolution mask feature be denoted by $F^{m}\in R^{C\times H\times W}$. The confidence map $C_{i}$ and boundary response map $B_{i}$ obtained at the i-th stage are upsampled to the same spatial resolution as $F^{m}$ and are denoted by $C_{i}^{↑}$ and $B_{i}^{↑}$, respectively. The structurally modulated mask feature at this stage is then defined as:

(19) $$F_{i}^{m}=F^{m}+\psi ([F^{m},C_{i}^{↑},B_{i}^{↑}])$$

After modulation by ψ(⋅), the output channel dimension of $F_{i}^{m}$ remains C. This residual enhancement form preserves the high-resolution semantic backbone while introducing boundary and reliability information as structural correction terms.

It should be noted that SCM does not establish a separate boundary prediction branch, nor does it introduce additional boundary supervision. Confidence maps are used here to distinguish between stable and uncertain regions, whilst boundary response maps provide clues regarding local structural changes. Both are incorporated into a shared high-resolution mask feature in the form of residual correction. Consequently, SCM is able to perform selective structural correction on elongated defects, regions with weak responses and ambiguous boundaries, whilst preserving the original high-resolution semantic information.

Once the stage-wise query representation $Q_{i}$ is obtained, the output of the current stage is generated by the stage prediction head:

(20) $${\hat{Y}}_{i}=H_{i}(Q_{i},F_{i}^{m})$$

where $H_{i}(\cdot )$ denotes the prediction head at the i-th stage, which consists of query normalization, query projection, and linear matching with mask features. Specifically, the query representation is first normalized by layer normalization, and a linear mapping is applied to predict the weight score of each query:

(21) $$w_{i}=Linear(LN(Q_{i})),w_{i}\in R^{N\times 1}$$

where $w_{i}$ represents the contribution weight of each query to the segmentation result at the current stage.

The normalized query representation is then projected into a mask embedding:

(22) $$E_{i}=MLP(LN(Q_{i})),E_{i}\in R^{N\times C}$$

Next, linear matching is performed between the mask embedding and the high-resolution mask feature along the channel dimension to obtain the mask response associated with each query:

(23) $$M_{i}=E_{i}\cdot Flatten(F_{i}^{m}),M_{i}\in R^{N\times HW}$$

which is further reshaped as $M_{i}\in R^{N\times H\times W}$.

Finally, the mask response maps of all queries are fused by weighted summation using the weight scores predicted by the classification branch, yielding the single-channel segmentation prediction at the current stage:

(24) $${\hat{Y}}_{i}=\sum_{n=1}^{N}\;w_{i}^{\left(n\right)}M_{i}^{\left(n\right)}$$

In this way, the prediction head can simultaneously exploit the semantic selection capability of queries and the spatial detail representation of high-resolution mask features, thereby producing more accurate stage-wise segmentation results.

### 3.5. Loss Function

Segmentation supervision is imposed on both the initial prediction and the predictions at all decoding stages. A combination of binary cross-entropy loss and IoU loss is adopted as the training objective. Let Y denote the ground-truth label. The segmentation loss at the i-th stage is defined as:

(25) $$L_{seg}^{\left(i\right)}=L_{BCE}^{\left(i\right)}+L_{IoU}^{\left(i\right)}$$

The binary cross-entropy loss is formulated as:

(26) $$L_{BCE}^{\left(i\right)}=-\frac{1}{\left|\Omega \right|}\sum_{u\in \Omega }\;\left[Y(u)log{\hat{Y}}_{i}(u)+(1-Y(u))log(1-{\hat{Y}}_{i}(u))\right]$$

where Ω denotes the set of image pixels. The corresponding IoU loss is defined as:

(27) $$L_{IoU}^{\left(i\right)}=1-\frac{\sum_{u}\;{\hat{Y}}_{i}(u)Y(u)}{\sum_{u}\;{\hat{Y}}_{i}(u)+\sum_{u}\;Y(u)-\sum_{u}\;{\hat{Y}}_{i}(u)Y(u)}$$

Because the network contains one initial prediction and three stage-wise predictions, a deep supervision strategy is adopted, and the overall optimization objective is defined as the sum of the losses from all prediction stages:

(28) $$L_{total}=\sum_{i=0}^{3}\;L_{seg}^{\left(i\right)}$$

Accordingly, the total loss can be further written as:

(29) $$L_{total}=\sum_{i=0}^{3}\;\left(L_{BCE}^{\left(i\right)}+L_{IoU}^{\left(i\right)}\right)$$

It should be noted that no additional auxiliary supervision terms are introduced specifically for TDM, RPR, or SCM. Instead, the entire network is optimized end-to-end under a unified multi-stage segmentation objective. This design keeps the training process concise while allowing for all modules to be jointly learned toward the final pixel-level defect segmentation objective, thereby improving the overall segmentation performance.

## 4. Experiments and Results Analysis

### 4.1. Experimental Setting

#### 4.1.1. Datasets and Annotation Processing

The effectiveness of the proposed method was evaluated on two primary steel surface defect datasets, ESDIs-SOD [53] and NEU-DET [54]. ESDIs-SOD contains 4800 images, including 3600 training images and 1200 test images, and provides pixel-level binary annotations directly. This dataset covers multiple typical steel surface defects, including scratches, welding line, water spot, foreign matter inclusion, inclusion, patches, roll printing, and oxide scale of the temperature system. Owing to its strong texture interference, large scale variation, and blurred boundaries, it is well suited for evaluating defect-region extraction under complex industrial backgrounds.

**Figure 5:**
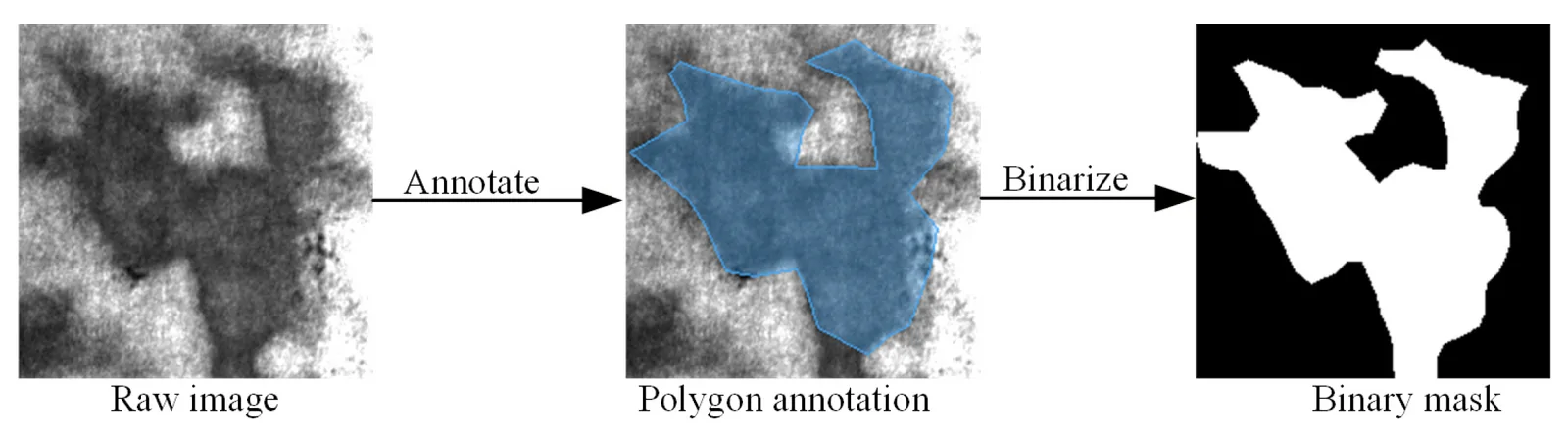
Illustration of the NEU-DET dataset processing procedure.

The NEU-DET release used in this study contains 4432 images with polygon annotations. The annotation files available in the present release contain three original label codes, namely In, Pa, and Sc, corresponding to inclusion, patches, and scratches, respectively. Individual images may contain more than one of these labels. The images are affected by illumination variation, material differences, and substantial intra-class appearance variation. Because this study focuses on binary segmentation between defect foreground and background rather than category recognition, all annotated defect polygons were merged into a single foreground class and converted into binary masks. Figure 5 illustrates the original images, polygon annotations, binary masks, and the annotation conversion process. NEU-DET was divided into training, validation, and test sets with a ratio of 8:1:1.

To examine whether binary category aggregation concealed an obvious performance decline for images containing a particular original defect label, the original labels were retained for a post hoc category-conditioned evaluation on the test set. Each subset contains all test images in which the corresponding label occurs, and the predictions are evaluated against the complete binary defect masks. Since images can contain multiple labels, these subsets are overlapping and are not interpreted as a multi-class segmentation benchmark.

It should be noted that the original annotations in NEU-DET are polygon-based annotations designed primarily for defect localization. In this study, the polygon annotations were rasterized into binary masks at the original image resolution, and all available annotated defect labels were merged into a single foreground class to match the binary segmentation setting. Therefore, the converted masks provide region-level defect delineation but may not represent exact physical defect boundaries in all cases. NEU-DET is consequently used in this study as a secondary benchmark for binary defect-mask segmentation rather than as the sole evidence for pixel-accurate boundary recovery. Since all compared methods were trained, tested, and evaluated using the same converted masks and evaluation protocol, the relative comparison remains fair. The results on ESDIs-SOD, which provides pixel-level defect annotations, are used as the primary evidence for evaluating fine-grained boundary and structural recovery.

To further assess zero-shot cross-dataset generalization, SD-saliency-900 [55] was introduced as an external test dataset. It contains 900 grayscale strip-steel surface images with pixel-level binary masks, including 300 inclusion samples, 300 patch samples, and 300 scratch samples. The original image resolution is 200 × 200. SD-saliency-900 was not used for model training, validation, checkpoint selection, or hyperparameter tuning. Instead, the models trained on ESDIs-SOD were directly tested on all 900 images. This setting evaluates the robustness of defect-mask prediction under changes in defect categories, surface textures, and image distributions.

#### 4.1.2. Evaluation Metrics

To quantitatively evaluate the performance of different methods on binary steel surface defect segmentation, mean absolute error (M) [56], mean F-measure ($mF_{\beta },\beta^{2}=0.3$) [57], weighted F-measure ($F_{\beta }^{w},\beta^{2}=1$) [58], S-measure ($S_{\alpha },\alpha =0.5$) [59], and mean E-measure ($mE_{\xi }$) [60] were adopted as the primary evaluation metrics. In addition, to provide a more intuitive comparison of the trade-off between precision and recall, precision–recall (PR) curves, F-measure curves, and E-measure curves were further plotted to comprehensively compare segmentation performance under different thresholds.

#### 4.1.3. Implementation Details

The proposed method was implemented in PyTorch 2.1.2, with PVTv2-B2 used as the backbone. Input images were uniformly resized to 384 × 384. The feature dimension was set to 64, and the number of queries was set to 16. During training, the Adam optimizer was employed with an initial learning rate of 8 × 10^−5^, and cosine annealing was used for learning-rate decay. The total number of training epochs was set to 150. The loss function consisted of binary cross-entropy loss and IoU loss, and deep supervision was imposed on both the initial prediction and all stage-wise predictions. Data augmentation mainly included random flipping and random rotation. The same preprocessing, training strategy, and evaluation protocol were used for both datasets. For reproducibility, all attention modules used eight heads. The RPR feed-forward network had a hidden dimension of 256, whereas the two-layer query-initialization decoder used a feed-forward dimension of 2048.

### 4.2. Comparison with State-of-the-Art Methods

To comprehensively evaluate the proposed method, nine representative segmentation methods were selected for comparison, namely UNet [61], PSPNet [62], DeepLabv3+ [63], UPerNet [64], SegFormer [65], DefectSAM [13], WPFormer [14], DEPANet [66], and MINet [67]. These methods cover classical encoder–decoder architectures, pyramid context modeling, dilated-convolution-based enhancement, multi-scale feature aggregation frameworks, Transformer-based segmentation models, and segmentation approaches strengthened by large-scale pretrained priors. In addition, WPFormer, DEPANet, and MINet were included as recent task-specific methods for industrial steel surface defect segmentation. WPFormer provides a strong Transformer-based comparison for modeling complex defect structures. DEPANet emphasizes edge-guided pyramid feature aggregation, and MINet is a lightweight multiscale interactive network designed for strip steel surface defect segmentation. Together with DefectSAM, these methods enable comparison across generic segmentation architectures, foundation-model-adapted segmentation, and recent industrial defect segmentation approaches. All methods were trained and evaluated using the same data split, binary mask annotations, and evaluation protocol, while retaining their respective model architectures and optimization strategies. This setting provides a more comprehensive and task-relevant basis for assessing local detail recovery, boundary preservation, global context modeling, and robustness to complex background textures.

#### 4.2.1. Results on the ESDIs-SOD Dataset

**(1)**  **Quantitative comparison**

Table 1 presents the quantitative comparison results on the ESDIs-SOD dataset. TDRPFormer achieved the best performance across all core metrics, with M=0.0185, $mF_{\beta }=0.8780$, $F_{\beta }^{w}=0.8824$, $S_{\alpha }=0.9070$, and $mE_{\xi }=0.9626$. Compared with SegFormer, which was the strongest conventional baseline, TDRPFormer improved $F_{\beta }^{w}$ by 0.1594 and reduced M by 0.0245. Compared with WPFormer, the strongest task-specific industrial defect segmentation baseline, TDRPFormer further improved $F_{\beta }^{w}$ by 0.0164 and reduced M by 0.0011. These results indicate that the proposed method not only localized defect regions more accurately but also provided better control of pixel-level error.

Among the baseline models, SegFormer yielded the strongest performance among conventional segmentation methods, indicating that hierarchical Transformer-based feature representation is advantageous for defect-region modeling in complex industrial scenarios. DefectSAM, as a comparison method incorporating large-scale pretrained priors, also achieved competitive performance, with $F_{\beta }^{w}$ = 0.8284, exceeding the conventional segmentation baselines. This result indicates that pretrained visual priors contribute positively to defect-region perception. However, DefectSAM remained inferior to WPFormer and TDRPFormer, particularly in terms of pixel-level error control and structural consistency.

WPFormer, DEPANet, and MINet provide task-specific comparisons for industrial steel surface defect segmentation. WPFormer achieved the strongest performance among these industrial baselines, with $F_{\beta }^{w}$ = 0.8660, followed by DEPANet with $F_{\beta }^{w}$ = 0.8490. MINet obtained $F_{\beta }^{w}$ = 0.8209 and $S_{\alpha }$ = 0.8820, indicating that its lightweight multiscale interaction design was less effective in handling complex background textures and fine defect structures in this dataset. Overall, the comparison shows that TDRPFormer maintains a clear advantage over recent industrial steel surface defect segmentation methods under the same evaluation protocol.

**Figure 6:**
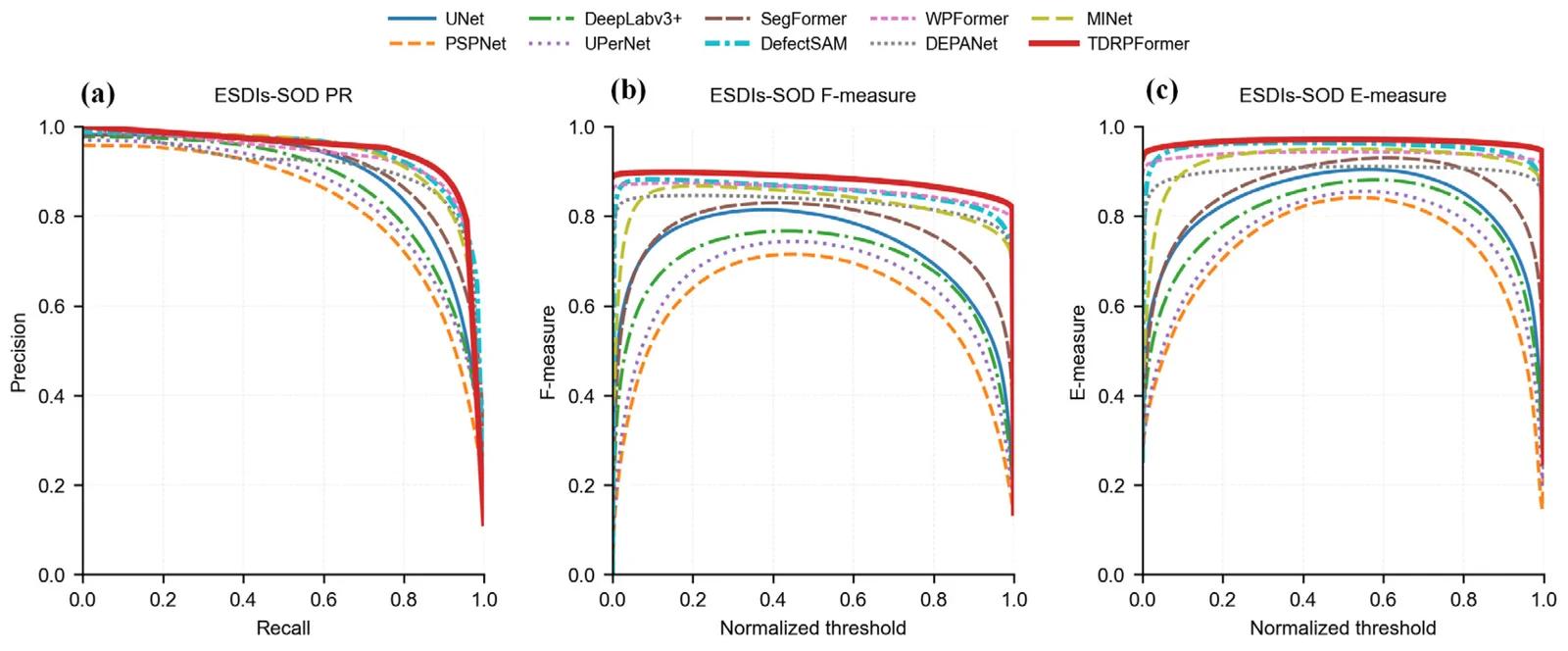
Curve comparison results on the ESDIs-SOD dataset: (**a**) PR curve, (**b**) F-measure curve, and (**c**) E-measure curve.

Figure 6 further shows the PR curves, F-measure curves, and E-measure curves of different methods on the ESDIs-SOD dataset. Overall, TDRPFormer formed the best envelope across all three types of curves, and its PR curve remained closest to the upper-right corner. This result shows that high precision was maintained as recall increased. In particular, the precision decay of TDRPFormer was substantially smaller than that of the competing methods in the high-recall regime, reflecting stronger suppression of false positives under complex backgrounds. Among the task-specific industrial defect segmentation methods, WPFormer remained the closest competitor to TDRPFormer, while DEPANet and MINet also provided stronger comparisons for complex steel surface defect segmentation than the conventional segmentation baselines.

The F-measure and E-measure curves further show that TDRPFormer maintained higher response values over most threshold intervals and exhibited a wider high-value plateau. This observation indicates lower sensitivity to threshold variation and better output stability. By contrast, the curves of PSPNet, DeepLabv3+, and UPerNet were generally lower and dropped earlier in the medium-to-high threshold range, suggesting that these methods were more prone to missed detections or discontinuous responses in low-contrast defect regions and under complex texture interference. As the strongest conventional baseline, SegFormer outperformed most CNN-based models overall, but still showed a noticeable precision drop in the high-recall region, indicating that background false responses were more easily introduced when broader defect coverage was pursued. The three curves of DefectSAM were all close to those of TDRPFormer, again showing the benefit of pretrained visual priors for defect-region modeling. However, DefectSAM still remained slightly below the proposed method over part of the threshold range, indicating that TDRPFormer retained a more stable advantage in foreground structural consistency and boundary refinement. The curves of WPFormer, DEPANet, and MINet further show that TDRPFormer maintained a more favorable balance between precision, threshold stability, and structural consistency under complex steel surface backgrounds.

**(2)**  **Qualitative comparison**

**Figure 7:**
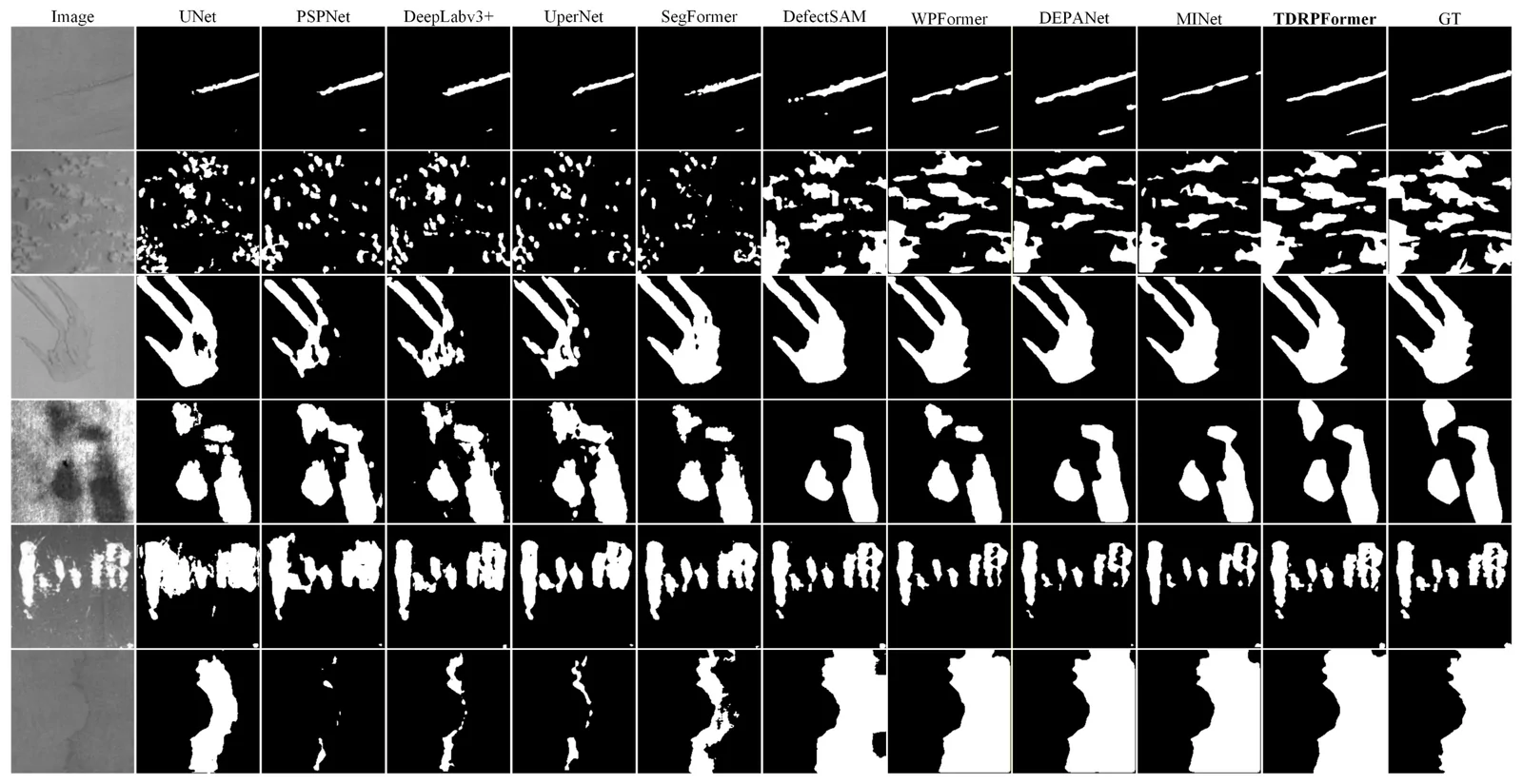
Representative segmentation examples of different models on the ESDIs-SOD dataset.

Figure 7 presents representative segmentation results of the different methods on the ESDIs-SOD dataset. For samples containing slender scratches, low-contrast targets, and blurred-boundary defects, the predictions of TDRPFormer were most consistent with the ground truth. Not only was the main defect body recovered more completely, but narrow structures and local accessory regions were also better preserved. By contrast, PSPNet, DeepLabv3+, and UPerNet showed different degrees of target fragmentation, boundary erosion, and local missed detections in several samples, indicating insufficient stability in their responses to fine-grained structures and weakly salient regions. UNet produced relatively complete predictions on some regular targets, but its ability to recover complex boundaries and small accessory defects remained limited. SegFormer localized the main target regions more accurately, but insufficient edge detail or local incompleteness was still observed in a few cases. WPFormer and DEPANet also recovered the main defect regions in several samples, but local boundary deviations and incomplete recovery of secondary structures were still observed under complex backgrounds. MINet produced reasonable masks for relatively salient defects, whereas its responses to weak, fragmented, or camouflage-like defect regions were less stable.

DefectSAM also showed a strong main-body detection ability in several challenging samples, but local region expansion, adhesion between adjacent defects, and scattered false positives could still be observed. WPFormer and DEPANet showed comparatively compact predictions in some samples, but their masks still exhibited local over-segmentation or incomplete boundary recovery in challenging cases. MINet was more likely to miss fine accessory regions or produce less complete responses for defects with weak contrast. By contrast, TDRPFormer preserved target completeness while suppressing background noise more effectively, yielding smoother mask boundaries and more compact structures. For example, in slender crack samples, both the primary structure and secondary details were retained. In large irregular defect samples, TDRPFormer also produced contours closer to the ground truth, while reducing internal holes and edge artifacts.

#### 4.2.2. Results on the NEU-DET Dataset

**(1)**  **Quantitative comparison**

Table 2 shows the quantitative comparison results on the NEU-DET dataset. TDRPFormer again achieved the best overall performance, with M=0.0210, $mF_{\beta }=0.8824$, $F_{\beta }^{w}=0.8858$, $S_{\alpha }=0.9017$, and $mE_{\xi }=0.9662$. Compared with UNet, which was the strongest conventional baseline on this dataset, TDRPFormer improved $F_{\beta }^{w}$ by 0.1007 and further reduced M by 0.0147. Compared with WPFormer, the strongest task-specific industrial defect segmentation baseline, TDRPFormer further improved $F_{\beta }^{w}$ by 0.0045 and reduced M by 0.0009. These results indicate that the proposed method not only provided better overall detection accuracy but also achieved better region-level mask agreement and preserved defect structures more effectively.

It is noteworthy that UNet became the strongest conventional baseline on NEU-DET. This result indicates that classical encoder–decoder architectures remain highly competitive in steel surface defect scenarios where texture patterns are relatively regular, and the foreground–background structure is comparatively stable. Even under such conditions, where local texture cues already provide relatively strong discriminative information, TDRPFormer still achieved further improvement. This observation indicates that the proposed feature representation mechanism is effective not only for global modeling in complex scenarios but also for enhancing fine-grained defect responses in regular industrial textures. DefectSAM also yielded competitive performance on NEU-DET, with $F_{\beta }^{w}=0.8413$ and $mE_{\xi }=0.9545$. This result suggests that prompt-based segmentation frameworks built on pretrained visual priors also have strong potential in steel surface defect segmentation. Nevertheless, clear gaps in the proposed method still remained in M, $F_{\beta }^{w}$, and $S_{\alpha }$, indicating that TDRPFormer was superior in pixel-level error control and foreground structure preservation under the common polygon-to-mask evaluation protocol.

WPFormer, DEPANet, and MINet provide further task-specific comparisons for industrial steel surface defect segmentation. WPFormer achieved the closest overall performance to TDRPFormer, with $F_{\beta }^{w}$ = 0.8813 and $mE_{\xi }$ = 0.9615. DEPANet also achieved competitive performance, with $F_{\beta }^{w}$ = 0.8776 and $S_{\alpha }$ = 0.8924, indicating the effectiveness of edge-guided pyramid aggregation for steel surface defect segmentation. MINet obtained $F_{\beta }^{w}$ = 0.8127 and $S_{\alpha }$ = 0.8759, suggesting that its lightweight multiscale interaction design was less effective in handling the detailed defect boundaries required by this dataset. Overall, the comparison shows that TDRPFormer maintained the best balance between pixel-level accuracy, structural consistency, and threshold-independent segmentation quality.

To further examine the effect of binary category aggregation, Table 3 reports a category-conditioned evaluation of TDRPFormer on the NEU-DET test set. The subsets were formed according to the original labels retained in the annotation files, including inclusion, patches, and scratches. Because a test image may contain more than one label, the subsets overlap, and the reported results reflect binary defect-mask segmentation performance for the images containing each label rather than mutually exclusive multi-class segmentation accuracy.

TDRPFormer maintained competitive performance across all three subsets. For inclusion-containing images, it achieved M = 0.0207, $mF_{\beta }$ = 0.8546, $F_{\beta }^{w}$ = 0.8588, $S_{\alpha }$ = 0.8880, and $mE_{\xi }$ = 0.9620. On the patches subset, the model obtained the highest mFβ, Fβw, Sα, and $mE_{\xi }$ values of 0.9198, 0.9199, 0.9085, and 0.9666, respectively, although its M value increased to 0.0286. On the scratches subset, TDRPFormer achieved M = 0.0200, $mF_{\beta }$ = 0.8771, $F_{\beta }^{w}$ = 0.8845, $S_{\alpha }$ = 0.9060, and $mE_{\xi }$ = 0.9692. These results show that the model retained stable binary defect-region extraction across images containing different original defect labels, while also revealing that the pixel-level error varied across subsets. This analysis does not replace a fully supervised multi-class segmentation evaluation, which would require reliable, mutually exclusive pixel-level masks for all defect categories.

**Figure 8:**
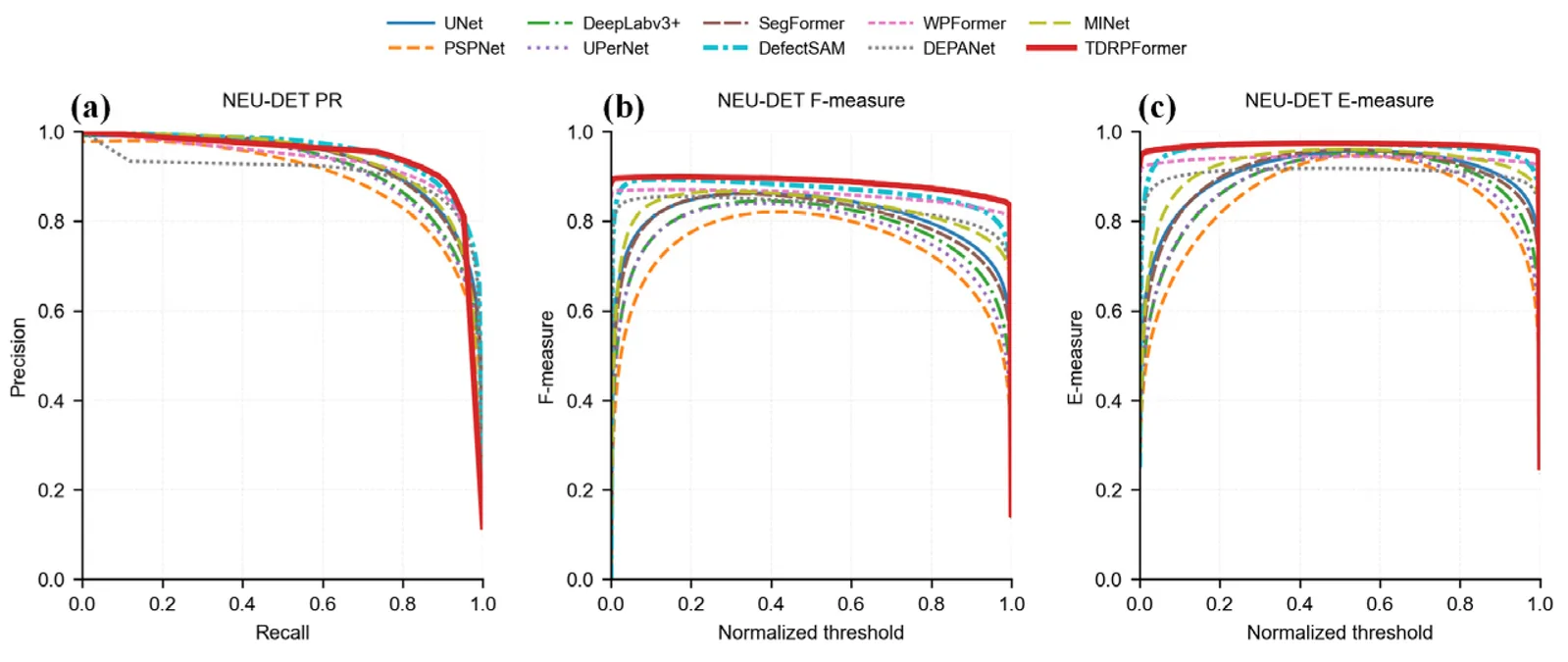
Curve comparison results on the NEU-DET dataset: (**a**) PR curve, (**b**) F-measure curve, and (**c**) E-measure curve.

Figure 8 further presents the PR curves, F-measure curves, and E-measure curves on the NEU-DET dataset. Similar to the results on ESDIs-SOD, TDRPFormer maintained the best overall trend across all three curves, and its PR curve remained closer to the upper-right corner. This result indicates that high detection precision was preserved while recall increased, reflecting a more concentrated foreground response to steel surface defect regions. In particular, TDRPFormer still exhibited relatively mild performance decay in the regime where both high precision and high recall were required, indicating more stable output characteristics under different binarization thresholds. Among the task-specific industrial defect segmentation methods, WPFormer remained the closest competitor to TDRPFormer, while DEPANet and MINet also provided representative comparisons under the NEU-DET setting.

The F-measure and E-measure curves show that TDRPFormer not only reached higher peaks but also maintained a broader high-value plateau over a wider threshold range, indicating lower sensitivity to threshold perturbation and better stability. Notably, DefectSAM also showed strong curve performance on NEU-DET, suggesting that pretrained priors substantially enhanced defect localization in regular industrial textures. However, its curves still remained slightly below those of the proposed method in the medium-to-high threshold range, indicating remaining limitations in boundary consistency and detail recovery. On the other hand, UNet and SegFormer performed relatively better among the conventional baselines, which is consistent with the characteristics of NEU-DET, where texture structures are more regular and local boundary information is more discriminative. By contrast, the curves of PSPNet, DeepLabv3+, and UPerNet were generally lower and dropped more rapidly in the high-threshold region, indicating that these methods remained limited in handling complex defect morphology and fine-grained boundary recovery. The curves of WPFormer, DEPANet, and MINet further show that TDRPFormer maintained a more favorable balance between precision, threshold stability, and structural consistency on NEU-DET.

**(2)**  **Qualitative comparison**

**Figure 9:**
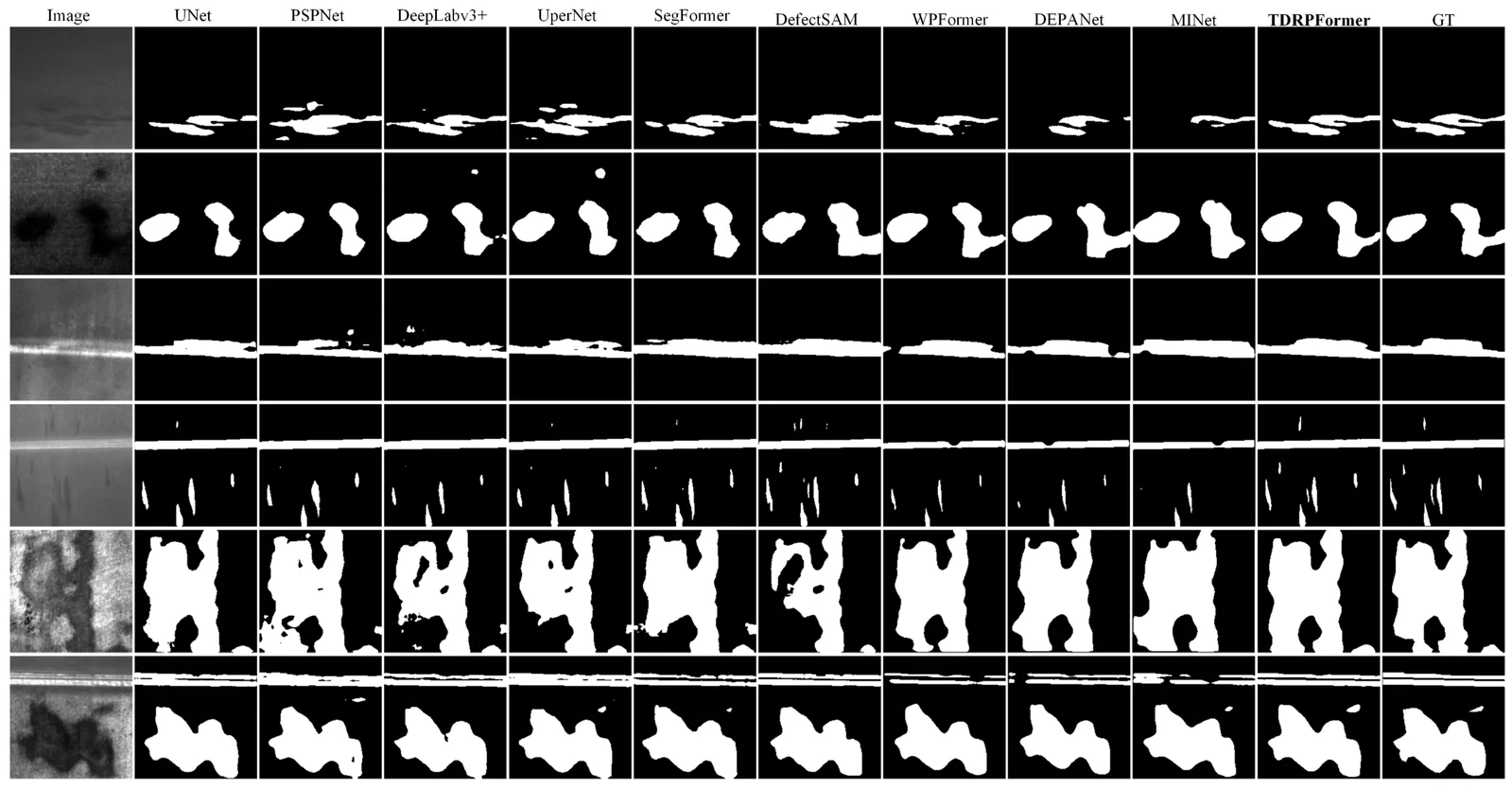
Representative segmentation examples of different models on the NEU-DET dataset.

Figure 9 shows representative qualitative results on the NEU-DET dataset. In samples containing slender cracks, small discrete defects, and irregularly shaped steel surface defects, the segmentation results of TDRPFormer were closest to the ground truth, especially in terms of boundary continuity, preservation of fine structures, and background suppression. For slender defect targets, several competing methods showed target fracture, local omission, or the loss of small accessory regions, whereas TDRPFormer recovered both the main contour and its associated structures more completely. For samples containing multiple defect instances, the conventional methods tended to produce excessive scattered noise, adhesion between defects, or fragmented regions. DefectSAM also showed a tendency toward over-expansion in some cases. WPFormer and DEPANet preserved the primary defect regions in several samples, but local contour deviations, incomplete secondary structures, or residual background responses were still observed in challenging cases. MINet generated reasonable masks for relatively clear defects, whereas its responses to small or weak defect structures were comparatively less stable. By contrast, TDRPFormer preserved regional completeness while suppressing irrelevant background responses more effectively.

Further inspection showed that, in samples with stronger texture interference or weaker defect boundaries, PSPNet, DeepLabv3+, and UPerNet were more likely to produce rough edges, local holes, or contour deviations. Although SegFormer and UNet preserved the main target regions relatively well in some cases, noticeable errors still remained for extremely fine structures and complex boundaries. WPFormer and DEPANet also achieved competitive qualitative results, but TDRPFormer showed more consistent boundary alignment and structural continuity across the representative samples. MINet retained the main regions in several cases but showed a relatively weaker recovery of fine details. By contrast, the defect masks generated by TDRPFormer were consistently closer to the ground truth in overall shape, boundary alignment, and local detail. These observations indicate that the proposed method not only localized target regions accurately but also preserved the geometric characteristics of defects more effectively.

#### 4.2.3. Zero-Shot Cross-Dataset Evaluation on the SD-Saliency-900 Dataset

To further evaluate cross-dataset generalization, all models trained on ESDIs-SOD were directly tested on the SD-saliency-900 dataset without any retraining or fine-tuning.

**(1)**  **Quantitative comparison**

Table 4 presents the zero-shot cross-dataset evaluation results on the SD-saliency-900 dataset. All models were trained on ESDIs-SOD and directly tested on SD-saliency-900 without retraining or fine-tuning. This external dataset contains strip-steel defects with different texture distributions, imaging conditions, and defect morphologies, providing a more challenging evaluation of cross-dataset robustness.

TDRPFormer achieved the best performance across all five metrics, with M = 0.0189, $mF_{\beta }$ = 0.8500, $F_{\beta }^{w}$ = 0.8482, $S_{\alpha }$ = 0.8920, and $mE_{\xi }$ = 0.9440. Compared to SegFormer, which was the strongest conventional baseline on this dataset, TDRPFormer improved $F_{\beta }^{w}$ by 0.2057, $S_{\alpha }$ by 0.0813, and $mE_{\xi }$ by 0.1314, while reducing M by 0.0396. These results indicate that the proposed method retained stronger defect-region discrimination and structural consistency when transferred from the ESDIs-SOD domain to a distinct strip-steel defect scenario.

Among the task-specific industrial defect segmentation methods, WPFormer was the closest competitor to TDRPFormer. Nevertheless, TDRPFormer further improved $mF_{\beta }$, $F_{\beta }^{w}$, $S_{\alpha }$, and $mE_{\xi }$ by 0.0029, 0.0026, 0.0019, and 0.0028, respectively, and reduced M by 0.0004. Although these improvements were modest, they were consistent across all reported metrics. DEPANet also achieved competitive performance, whereas MINet showed a more evident reduction in $F_{\beta }^{w}$ and $mE_{\xi }$, suggesting that lightweight multiscale interaction alone was less robust to the changed background texture distribution. Overall, the external evaluation indicates that TDRPFormer maintained stable zero-shot generalization across different steel surface defect datasets.

**(2)**  **Qualitative comparison**

**Figure 10:**
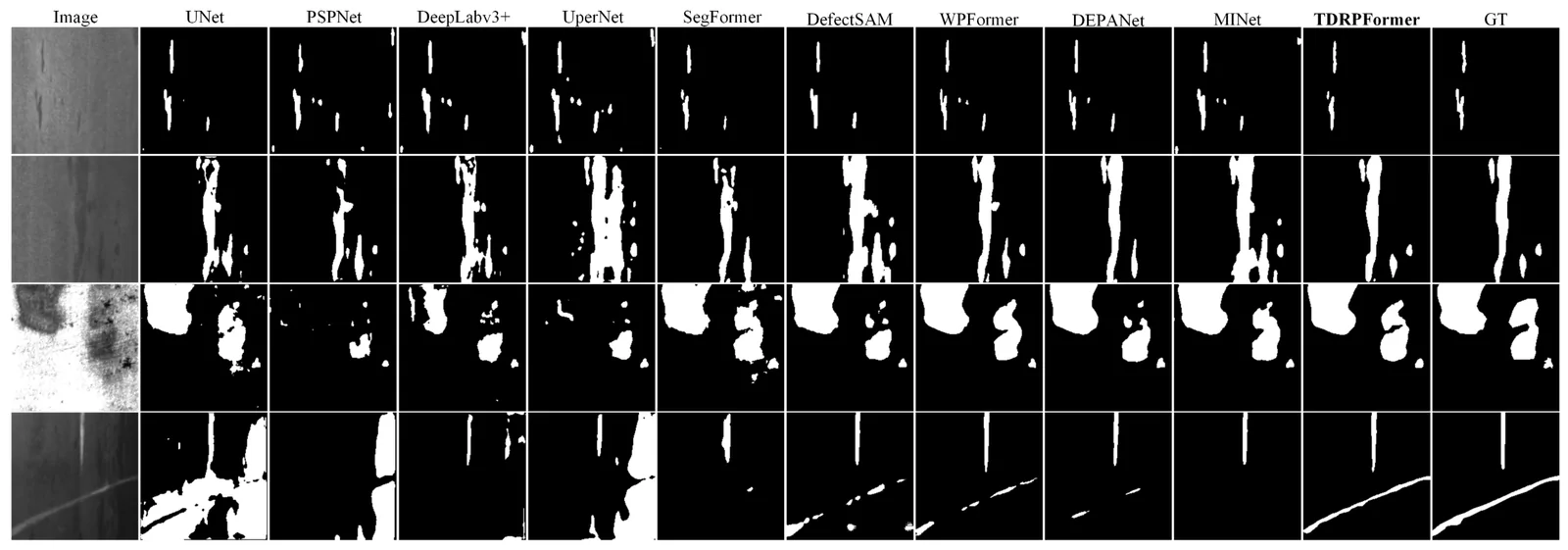
Representative zero-shot cross-dataset segmentation examples on the SD-saliency-900 dataset. All models were trained on ESDIs-SOD and directly tested on SD-saliency-900 without retraining or fine-tuning.

Figure 10 presents representative zero-shot segmentation results on the SD-saliency-900 dataset. Although all models were trained only on ESDIs-SOD, TDRPFormer produced predictions that were generally closer to the ground truth under the changed strip steel texture distribution. For samples containing slender defects and multiple separated target regions, TDRPFormer preserved the main defect bodies while retaining small accessory regions more completely. By contrast, UNet, PSPNet, DeepLabv3+, and UPerNet showed different degrees of target fragmentation, local omission, or scattered false responses.

In samples with irregular defect shapes and strong background interference, several competing methods exhibited region expansion, incomplete contours, or residual noise around the target areas. SegFormer, DefectSAM, WPFormer, and DEPANet recovered the main defect regions relatively well in several cases, but local deviations in defect extent or fine structural details could still be observed. MINet produced reasonable predictions for relatively salient regions, whereas its responses to weak or elongated defects were less stable. By contrast, TDRPFormer maintained more compact masks and better correspondence with the ground truth, particularly for the long curved defect in the last sample. These qualitative observations are consistent with the quantitative results in Table 3, further indicating that TDRPFormer retained stable defect-region modeling under the cross-dataset distribution shift.

### 4.3. Ablation Studies

To further validate the effectiveness and design rationality of the key components in TDRPFormer, ablation studies were conducted from three perspectives: the contribution of core modules, alternative design strategies, and intermediate response behavior. Unless otherwise specified, all experiments were performed on both ESDIs-SOD and NEU-DET using the same data split, training strategy, and evaluation metrics as those adopted in the main experiments. To assess the stability of the ablation results, each configuration was independently trained three times using different random seeds under the same data split, training schedule, and evaluation protocol. The reported values are presented as the mean ± standard deviation over the three runs.

#### 4.3.1. Contribution of Core Modules

To systematically assess the effect of each module on the overall performance, ablation experiments were first carried out on the three core components of TDRPFormer, namely TDM, RPR, and SCM. In the baseline model, the backbone network, feature pyramid fusion, multi-stage prediction head, and query initialization mechanism were retained, whereas TDM, RPR, and SCM were removed. Defect segmentation was then completed using only the conventional feature interaction and mask prediction pipeline. The corresponding ablation results are reported in Table 5.

As shown in Table 5, the complete TDRPFormer achieved the best performance across all metrics on both datasets, indicating that TDM, RPR, and SCM provide stable and complementary gains. On ESDIs-SOD, for example, the full model reduced M from 0.0308 to 0.0185, increased $F_{\beta }^{w}$ from 0.7812 to 0.8824, and improved $S_{\alpha }$ from 0.8728 to 0.9070 relative to the baseline. These results indicate that prediction error was reduced while regional consistency and structural similarity were substantially enhanced. Similar improvements were also observed on NEU-DET, where M decreased from 0.0304 to 0.0210 and $F_{\beta }^{w}$ increased from 0.8217 to 0.8858, further confirming the robustness of the proposed method across different industrial defect scenarios. Moreover, the relatively small standard deviations across the repeated runs indicate that the performance improvements and complementary trends of the three modules were stable under different random initializations.

Among the individual modules, TDM contributed the largest gain. On ESDIs-SOD, introducing TDM alone increased $F_{\beta }^{w}$ from 0.7812 to 0.8316 and improved $mE_{\xi }$ from 0.9254 to 0.9461. On NEU-DET, $F_{\beta }^{w}$ was also increased from 0.8217 to 0.8511. These results indicate that TDM effectively suppresses complex background and pseudo-texture interference, thereby improving the concentration of model responses on true defect regions. By comparison, introducing RPR or SCM alone also produced stable improvements, but the gains were smaller than those brought by TDM. This finding suggests that background texture suppression remains a key prerequisite for performance improvement in industrial surface defect segmentation.

Further examination of the dual-module combinations shows that both TDM + RPR and TDM + SCM clearly outperformed their corresponding single-module settings. The former was slightly superior in $F_{\beta }^{w}$, whereas the latter performed better in $S_{\alpha }$ and $mE_{\xi }$. This pattern indicates that RPR contributes more to discriminative aggregation of defect regions, whereas SCM is more beneficial for preserving boundary continuity and structural integrity. By contrast, although RPR + SCM outperformed the single-module settings, its overall performance still remained inferior to the combinations containing TDM. This observation further suggests that TDM provides a cleaner and more stable feature basis for subsequent region routing and structural modulation. Overall, the three modules improve model performance from the complementary perspectives of background suppression, region modeling, and structural correction, and their joint use yields the best results.

#### 4.3.2. Analysis of Texture-Debiasing and Prediction-Conditioned Cues

The rationality of the internal design choices in TDRPFormer was further investigated from the perspectives of texture-debiasing and gating strategies, prediction-conditioned cues, query configuration, prototype configuration, and the interaction and modulation forms used in RPR and SCM. Unless otherwise specified, only the examined factor was changed, while the remaining architecture, training schedule, and evaluation protocol were kept identical to those of the full model. The corresponding results are reported in Table 6, Table 7, Table 8, Table 9 and Table 10.

With regard to texture-debiasing and gating strategies, Table 6 shows that all simplified variants improved segmentation performance to some extent, but their overall performance remained inferior to that of the complete TDM. On ESDIs-SOD, for example, $F_{\beta }^{w}$ gradually increased from 0.8398 for Identity to 0.8538 for Concat-Conv, with Direct-Add lying in between. This trend indicates that simple feature fusion can introduce useful auxiliary information, but its ability to suppress complex background textures remains limited. When Gate-Residual was adopted, $F_{\beta }^{w}$ further increased to 0.8672 and $S_{\alpha }$ improved to 0.9049, indicating that selective residual attenuation is more effective than direct feature fusion. Nevertheless, its performance was still lower than that of the complete TDM. The proposed TDM achieved the best results on both datasets, with M/$F_{\beta }^{w}$/$S_{\alpha }$ of 0.0185/0.8824/0.9070 on ESDIs-SOD and 0.0210/0.8858/0.9017 on NEU-DET. These results indicate that confidence-guided prototype estimation, adaptive gating, and feature fusion jointly contribute to effective texture debiasing. The repeated-run results further show that the superiority of the complete TDM over the simplified alternatives was consistently observed across different random seeds.

**Figure 11:**
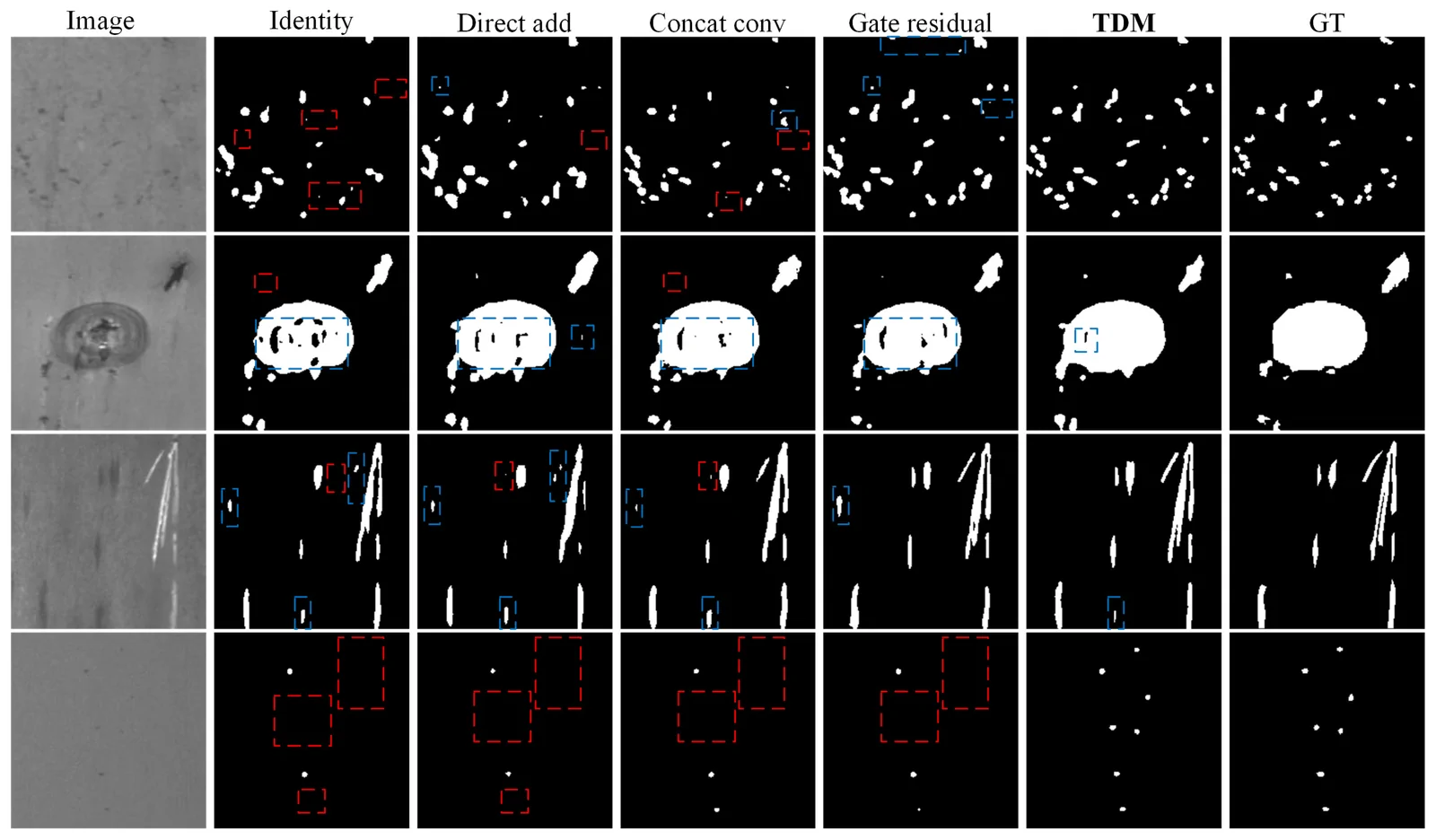
Visual comparison of different TDM replacement strategies. Red and blue boxes denote missed-detection and false-positive regions, respectively.

Figure 11 further compares the segmentation results under different TDM strategies. Without texture debiasing, the model tended to produce strong pseudo-responses in complex background regions, thereby introducing obvious false positives. After simple replacement strategies such as direct addition or concatenation followed by convolution were used, part of the background noise was alleviated, but missed detections and local adhesion still remained near the low-contrast regions and fine-grained boundaries. By contrast, the complete TDM achieved a better balance between complex textures and real defects. It not only suppressed background interference more effectively but also preserved the structural integrity of the defect body more faithfully.

#### 4.3.3. Effects of Confidence and Boundary-Response Cues

To separately examine the contribution of prediction-conditioned cues, the confidence map and boundary-response map were independently disabled while keeping the network architecture unchanged. Specifically, when the confidence cue was removed, the confidence map was replaced with a spatially uniform map, so that no pixel-wise reliability difference was provided to TDM, RPR, or SCM. When the boundary cue was removed, the boundary-response map was set to zero. The results are reported in Table 7.

As shown in Table 7, removing either prediction-conditioned cue led to a consistent performance decrease on both datasets. On ESDIs-SOD, disabling the confidence cue increased M from 0.0185 to 0.0206 and reduced Fβw from 0.8824 to 0.8709. This result indicates that pixel-wise reliability estimation helps TDM identify more trustworthy normal-texture regions and helps the subsequent routing process reduce interference from uncertain responses. Removing the boundary cue also degraded the performance, with M and Fβw changing to 0.0198 and 0.8767, respectively. Although this degradation was smaller than that caused by removing the confidence cue, it still indicates that boundary information contributes to the preservation of slender structures and blurred contours.

When both cues were removed, the performance drop became more pronounced, with M/Fβw/Sα changing to 0.0222/0.8614/0.9012 on ESDIs-SOD and 0.0239/0.8686/0.8974 on NEU-DET. These results indicate that confidence estimation and boundary response provide complementary reliability and structural information. The former mainly improves the selection of reliable regions and background suppression, whereas the latter provides additional structural constraints for boundary recovery. The same performance ordering was maintained over the repeated runs, indicating that the contributions of confidence estimation and boundary response were not caused by a particular random initialization.

#### 4.3.4. Effects of Query and Prototype Configurations

The numbers of decoder queries and reliable prototypes were further evaluated independently. For the query-number study, the number of reliable prototypes was fixed at $N_{p}$ = 16, while the number of decoder queries N was varied. For the prototype-number study, N was fixed at 16 and only $N_{p}$ was changed. In this way, the influence of the two factors could be separately assessed.

Regarding the setting of the query number, Table 8 shows that the model performance first improved and then gradually stabilized as the number of queries increased. When N=8, the performance on both datasets was slightly lower, indicating that a small number of queries limited the representation of diverse defect patterns. When N was increased to 16, the best overall performance was achieved, suggesting that this setting provided a favorable balance between representation capacity and training stability. When the number of queries was further increased to 32 and 64, no further improvement was observed, and M even increased slightly. This result indicates that too many queries may introduce redundant representations and increase optimization difficulty. Therefore, N=16 was finally adopted as the default setting. The repeated-run statistics also show that N = 16 consistently achieved the best balance between representation capacity and optimization stability.

Table 9 reports the effect of the number of reliable prototypes when Q was fixed at 16. A small number of prototypes limited the representation of diverse defect patterns and background structures. When N = 4, the M/$F_{\beta }^{w}$/$S_{\alpha }$ on ESDIs-SOD were 0.0205/0.8686/0.9044, indicating that the compact representation was insufficient for describing heterogeneous defect regions.

Increasing N from 4 to 16 consistently improved the segmentation performance. In particular, $F_{\beta }^{w}$ increased from 0.8686 to 0.8824 on ESDIs-SOD and from 0.8761 to 0.8858 on NEU-DET. This result indicates that a sufficient number of prototypes is required to preserve diverse regional structures during query refinement. However, further increasing K to 32 did not bring additional gains. The slight performance decrease suggests that an overly large prototype set may introduce redundant regional representations and weaken routing efficiency. Therefore, N = 16 provided the most favorable balance between representation capacity and compactness. The small variations across the repeated runs further support the selection of $N_{p}$ = 16 as the default prototype configuration.

Table 10 reports the effect of different interaction and modulation forms used in RPR and SCM. For the interaction pattern in RPR, using only contextual interaction or only prototype interaction both yielded certain gains, but the joint use of the two achieved the best performance. On ESDIs-SOD, Context + Prototype reduced M from 0.0227 to 0.0185 and increased $F_{\beta }^{w}$ from 0.8581 to 0.8824 compared with Context only, and it also showed a clearer advantage over Prototype only. These results indicate that contextual information helps provide global semantic constraints, whereas prototype information strengthens the aggregation of local defect patterns. Their combination, therefore, improves query updating more effectively. For SCM, both Identity and Concat-Conv preserved structural information to a certain extent, but the complete residual modulation strategy still achieved the best results. This result indicates that, after the main body information of the original mask feature is retained, gradual refinement using confidence and boundary-response cues is more beneficial for recovering slender structures and complex boundaries. The repeated-run results show that the performance advantage of the joint contextual and prototype interaction in RPR, as well as that of residual modulation in SCM, remained consistent across different random seeds.

**Figure 12:**
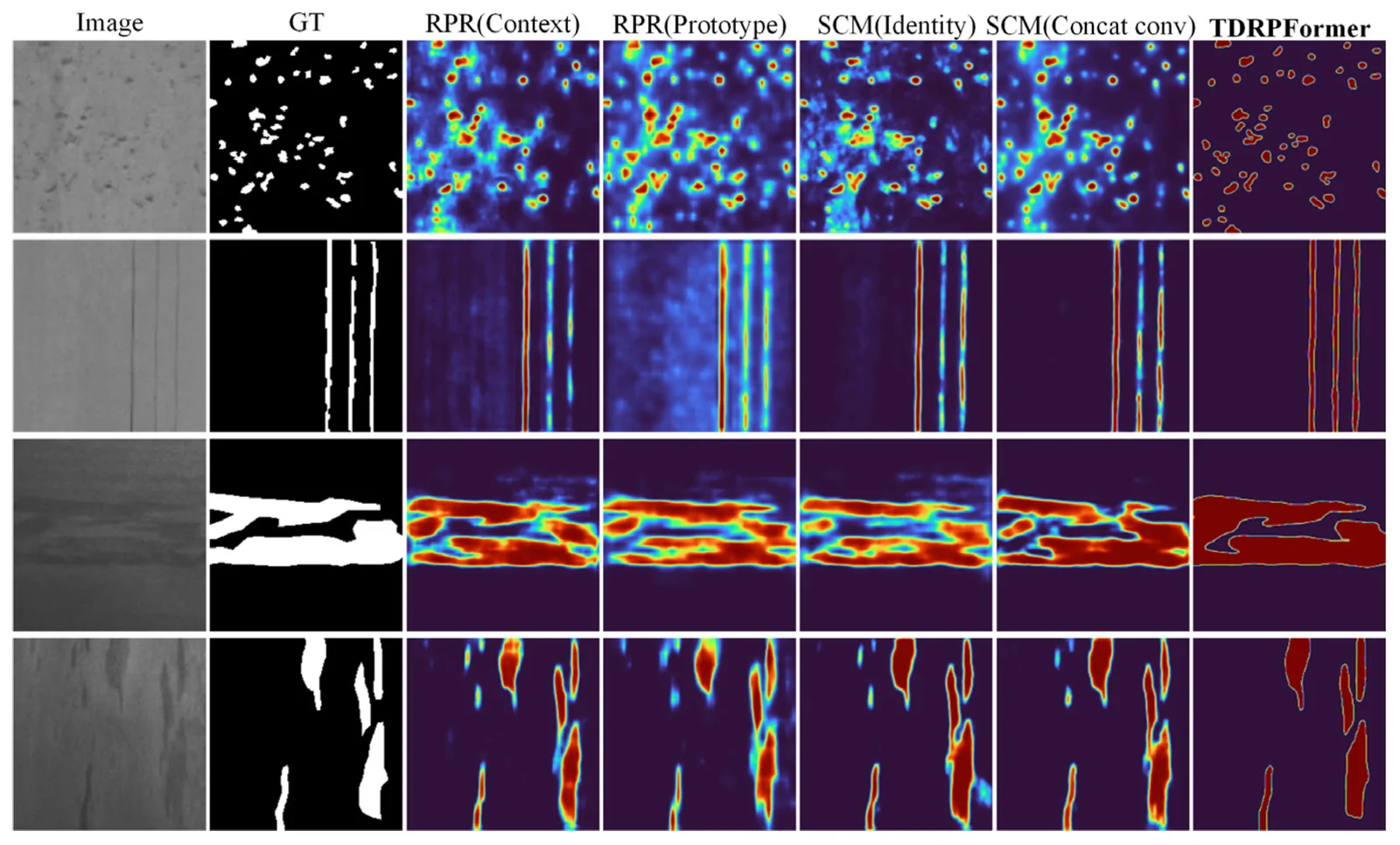
Comparison of response heatmaps under different interaction and modulation forms.

Figure 12 further presents comparisons of response heatmaps under different interaction and modulation forms. When only a single interaction path was used, the model response was relatively dispersed and was more easily disturbed by background regions and local noise, leading to less concentrated high responses in true defect regions. By contrast, after contextual interaction and prototype interaction were combined, the responses were more stably focused on the target regions, and more continuous high-response distributions were formed around the irregular boundaries and slender structures. For the structural modulation component, simple fusion strategies enhanced local responses to a certain degree, but the corresponding heatmaps still exhibited blurred boundaries and local discontinuities. In contrast, the residual modulation strategy further strengthened the continuity and consistency of the boundary regions while preserving the response intensity of the main body, making the final prediction closer to the ground truth. These observations provide intuitive support for the rationality of the designs of RPR and SCM from the perspective of intermediate responses and also explain the performance improvements reported in Table 10.

#### 4.3.5. Qualitative Analysis of Module-Specific Error Reduction

**Figure 13:**
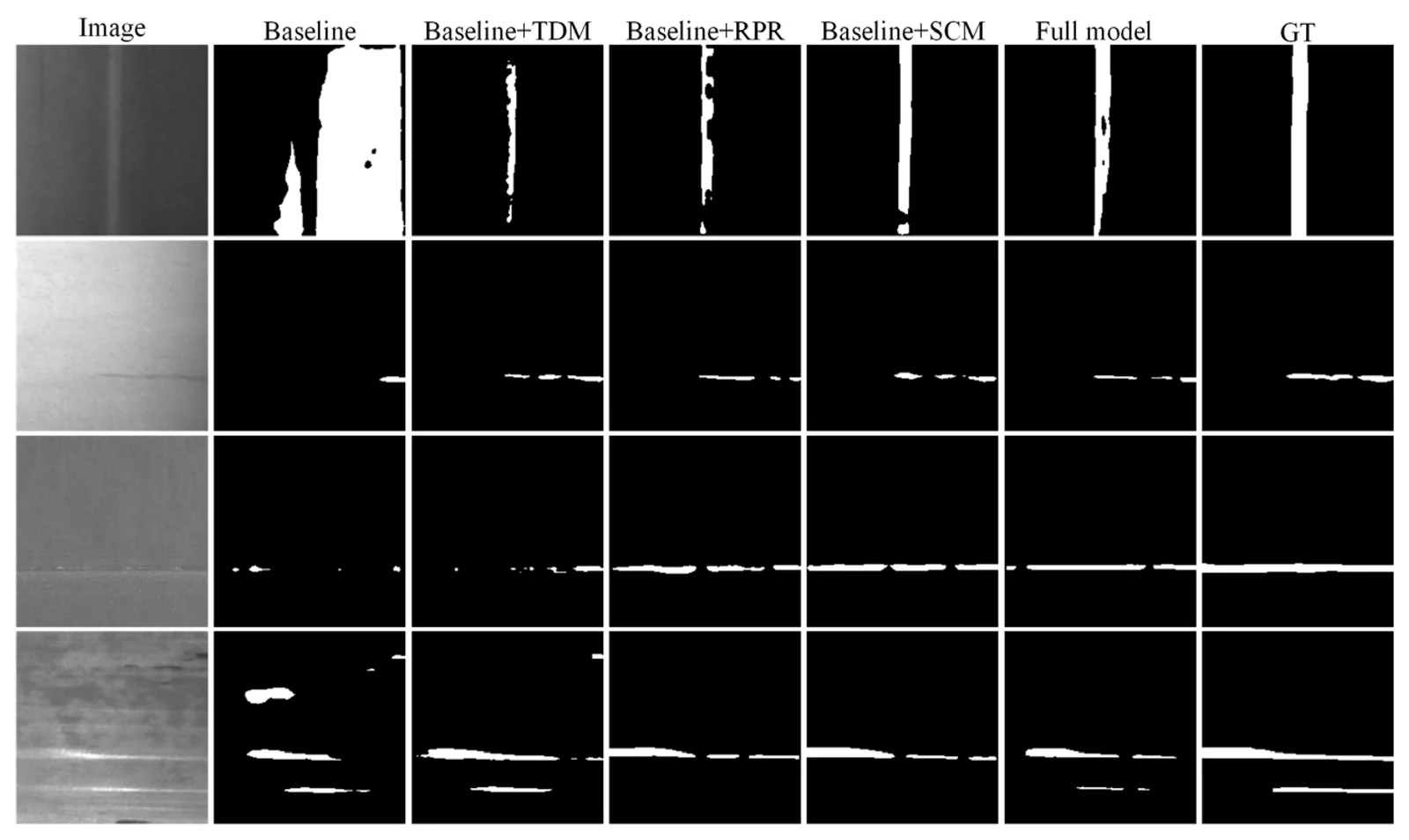
Qualitative comparison of ablated variants on representative challenging samples. The examples illustrate false positives caused by background textures, missed small defects, boundary shrinkage, and broken slender structures.

To further establish the connection between the quantitative ablation results and representative failure scenarios, Figure 13 compares the baseline model, three single-module variants, and the full TDRPFormer on the same challenging samples. The selected examples include false positives caused by background textures, missed small or weak defects, boundary shrinkage, and broken slender structures. This comparison makes it possible to identify the specific error types that are mitigated by each module.

As shown in the first row of Figure 13, the baseline model produced a large false-positive region under strong background texture interference. After TDM was introduced, the irrelevant background response was substantially suppressed, and the prediction became more concentrated on the true defect structure. This observation indicates that confidence-guided normal-texture debiasing effectively reduces the dominance of background patterns. In the second row, where the defect response was weak and only a small region was visible, the baseline model missed most of the target. The RPR variant recovered a more complete defect response, indicating that reliable prototype routing improves the aggregation of discriminative regional information and alleviates missed detections caused by dispersed or weak activations.

The third and fourth rows further show the effects of structural modeling on slender and low-contrast defects. The baseline predictions were fragmented or strongly incomplete, whereas the SCM variant improved contour continuity and reduced local shrinkage. Although the individual variants each alleviated a specific error type, the full model achieved the most consistent results across all representative samples. It simultaneously suppressed background-induced false positives, recovered weak defect regions, and preserved the continuity of slender structures, producing masks that were closest to the ground truth. These qualitative observations are consistent with the module-combination results reported in Table 5, further supporting the complementary roles of TDM, RPR, and SCM.

#### 4.3.6. Prototype Correspondence Analysis

**Figure 14:**
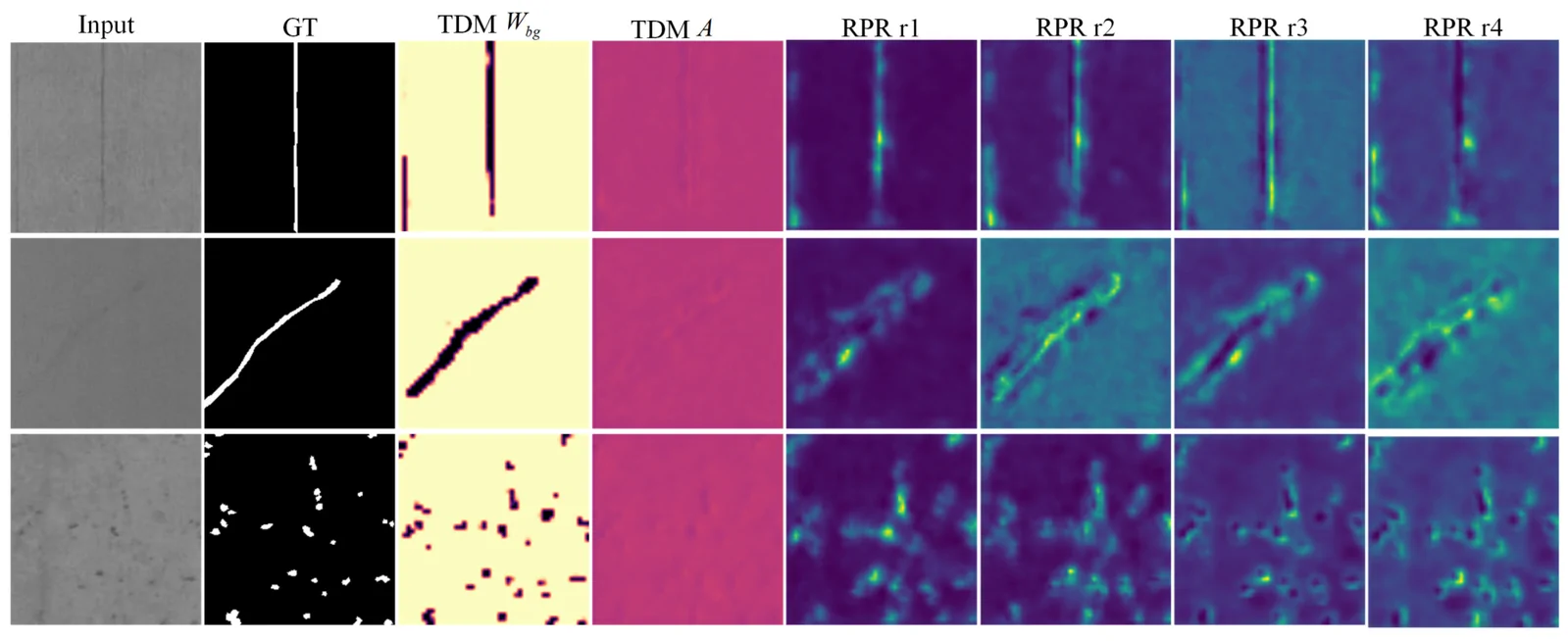
Visualization of TDM and RPR correspondence at the third refinement stage on representative ESDIs-SOD test images. The TDM background weight map $W_{bg}$ is estimated from prediction confidence and background probability, and A denotes the spatial attenuation gate. For each image, the four displayed RPR assignment maps are ranked by their foreground concentration ratio ρ, defined as the proportion of prototype assignment mass located within the ground-truth defect region. The displayed maps therefore represent the most defect-concentrated prototypes rather than all routed prototypes.

To provide direct evidence for the correspondence of the intermediate representations, Figure 14 visualizes the TDM background weights, attenuation maps, and RPR prototype assignment maps at the third refinement stage. In all three rows, the TDM background weight maps exhibited relatively high responses in the surrounding normal-texture regions, whereas the annotated defect structures were assigned lower background weights. This pattern can be observed for the slender defect in the first row, the elongated curved defect in the second row, and the scattered small defects in the third row. The attenuation maps further show that the background prototype was modulated spatially rather than subtracted uniformly from all locations.

For RPR, the foreground concentration ratio ρ was calculated for each prototype assignment map. The four maps with the highest ρ values are displayed for each row. In the first row, the most defect-concentrated prototype achieved ρ = 0.0826, whereas the foreground occupied only 0.0176 of the image area. Similarly, the highest ρ values were 0.0353 and 0.0562 in the second and third rows, compared with foreground-area ratios of 0.0251 and 0.0303, respectively. These observations indicate that some routed prototypes preferentially concentrated on the main defect body or local defect structures. At the same time, not every prototype was defect-specific, which is consistent with the role of RPR as prediction-conditioned structural routing rather than explicit defect-prototype supervision.

#### 4.3.7. Confidence-Consistency Analysis

The confidence maps used in TDRPFormer are deterministic entropy-derived reliability cues rather than calibrated uncertainty estimates. To examine whether these cues are consistent with prediction reliability, a quantitative analysis was conducted on the complete ESDIs-SOD test set using the prediction prior and TDM background weights at the third refinement stage. As reported in Table 11, the mean confidence of the correctly predicted pixels was 0.9925, which was substantially higher than that of incorrectly predicted pixels (0.8124). Correspondingly, the mean normalized entropy increased from 0.0075 for correct pixels to 0.1876 for incorrect pixels. These results indicate that pixels associated with prediction errors were more likely to exhibit uncertain responses.

Further stratified analysis showed a consistent relationship between confidence and pixel-level error. The error rates for the confidence intervals C<0.50, 0.50≤C<0.75, 0.75≤C<0.90, and C≥0.90 were 0.4754, 0.4441, 0.4054, and 0.0220, respectively. Therefore, the entropy-derived confidence cue provided a meaningful reliability indicator for distinguishing stable and ambiguous prediction regions.

The background-weight analysis further showed that 0.9851 of the total TDM background-weight mass was assigned to ground-truth background regions, whereas the background occupied 0.8848 of the evaluated spatial locations. This corresponded to a background-weight purity enrichment of 1.1133. Thus, the confidence-weighted background construction preferentially emphasized normal texture regions instead of uniformly aggregating all spatial features.

**Figure 15:**
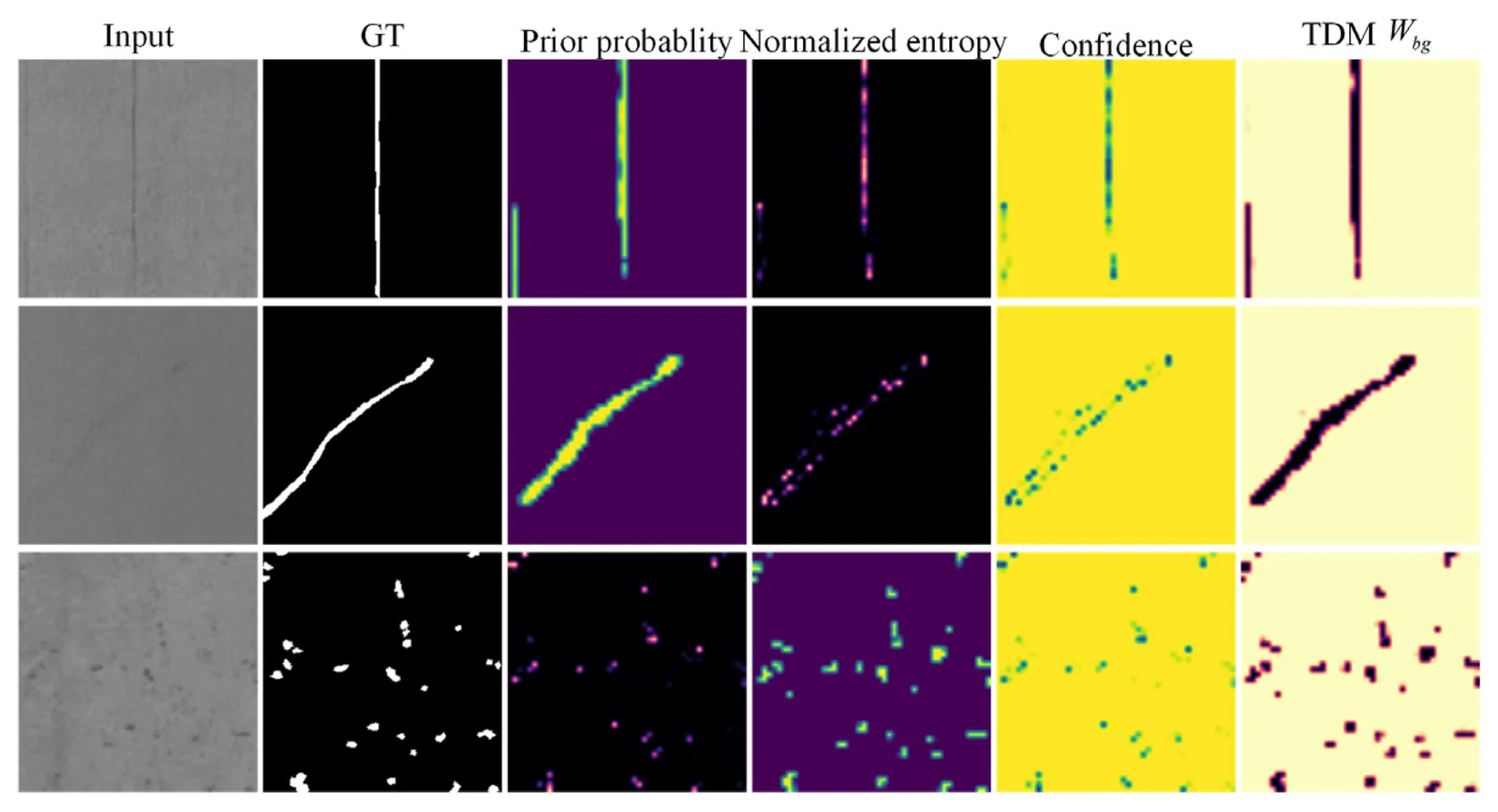
Visualization of the deterministic confidence cue and TDM background weighting at the third refinement stage. From left to right: input image, GT, prior probability map, normalized predictive entropy map, confidence map, and TDM background-weight map. The confidence cue is defined as $C=1-\frac{H\left(p\right)}{log2}$, where $H\left(p\right)$ denotes binary predictive entropy. High background weights are assigned preferentially to reliable background regions for normal-texture prototype construction.

Figure 15 provides representative visual evidence of this relationship. In the first, second, and third rows, the entropy maps show elevated responses mainly around defect structures, weak target regions, or ambiguous local areas, while the corresponding confidence maps show reduced values at these locations. The TDM background-weight maps further suppress these uncertain or foreground-dominant regions and retain stronger responses in the surrounding normal-texture areas. These results support the use of confidence as a deterministic routing cue for normal-texture prototype construction and subsequent feature modulation.

### 4.4. Complexity and Efficiency Analysis

While segmentation performance is a primary concern, parameter scale, computational complexity, and inference efficiency are also important considerations in industrial defect detection scenarios. To this end, the number of parameters, FLOPs, single-image inference latency, and inference speed of the different methods were further evaluated, and the results are reported in Table 12.

In terms of parameter scale, SegFormer exhibited the lightest architecture, with only 3.72 M parameters, whereas UPerNet and DefectSAM were relatively large, reaching 66.40 M and 93.74 M parameters, respectively. By comparison, the proposed TDRPFormer contains 26.74 M parameters, which is substantially lower than those of PSPNet, DeepLabv3+, UPerNet, and DefectSAM, and is also slightly lower than that of UNet. These results indicate that the performance improvement of the proposed method was not obtained through excessive parameter accumulation and that reasonable parameter efficiency was still maintained overall.

In terms of computational cost, TDRPFormer required 15.88G FLOPs, which is markedly lower than those of traditional encoder–decoder structures, such as UNet, PSPNet, DeepLabv3+, and UPerNet, and is exceeded only by the more lightweight SegFormer. This finding indicates that, although multi-stage prediction, prototype routing, and structural modulation were introduced, the overall forward computation remained within a relatively controllable range. By contrast, DefectSAM reached 488.24G FLOPs because of its higher input resolution and larger foundation model, leading to a computational cost far higher than that of the other methods.

In terms of inference efficiency, UNet achieved the highest inference speed, reaching 147.21 FPS, while SegFormer, PSPNet, DeepLabv3+, and UPerNet also exhibited substantially lower latency than TDRPFormer. The proposed model required 56.47 ms per image, corresponding to 17.71 FPS. This result indicates that the lower FLOPs of TDRPFormer did not translate directly into higher practical throughput. Although the computational cost of its individual operations remained moderate, the sequential three-stage refinement process, repeated cross-attention, prototype aggregation, and high-resolution mask modulation introduced additional memory-access and synchronization overhead.

Therefore, TDRPFormer should not be regarded as the fastest or a strictly real-time segmentation model among the compared methods. Its practical advantage lies in achieving improved segmentation accuracy with a moderate parameter scale and lower FLOPs than several heavy CNN-based baselines, at the cost of increased inference latency. This trade-off may be suitable for industrial inspection settings in which segmentation reliability is prioritized over maximum throughput, whereas further acceleration will be required for latency-critical deployment scenarios.

### 4.5. Failure Cases and Limitations

**Figure 16:**
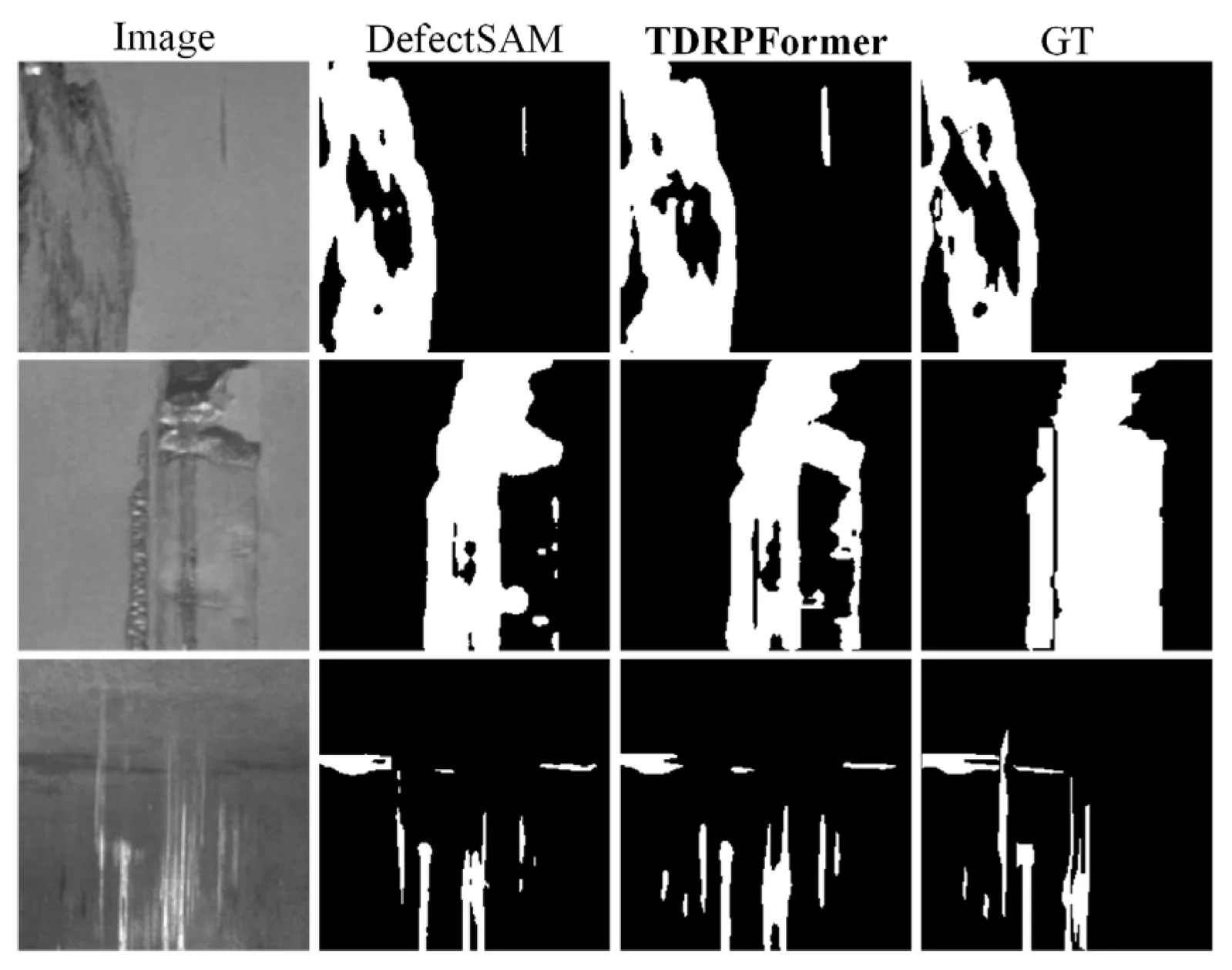
Representative failure cases and limitation analysis. Visual comparisons of DefectSAM, TDRPFormer, and ground truth (GT) are presented for several difficult samples.

Although TDRPFormer achieved the best quantitative results on both ESDIs-SOD and NEU-DET and showed strong background suppression and structural recovery in most samples, certain limitations still remained in several extremely challenging scenarios. To more objectively define the applicability boundary of the proposed method, several representative failure cases are presented in Figure 16, together with visual comparisons against the representative baseline DefectSAM.

As shown in Figure 16, the first type of sample contains defect regions with irregular shapes, blurred boundaries, and obvious fluctuations in internal gray-level distribution. In such cases, although TDRPFormer recovered the main contour more effectively than DefectSAM, a certain degree of boundary deviation could still be observed at local edges. This result indicates that the model remains limited in depicting fine contours when the internal texture of the target varies substantially and the boundary contrast is insufficient.

The second type of sample is characterized by slender targets located very close to surrounding structured backgrounds. In such scenarios, the background texture is highly similar to the defect region and can easily interfere with the discrimination process. It can be observed that, although TDRPFormer was superior to DefectSAM in overall structural recovery, incomplete segmentation and contour shrinkage still occurred in local regions. This finding suggests that fine-grained boundary modeling under complex backgrounds remains improvable.

The third type of sample corresponds to difficult defects that are low in contrast, slender in morphology, and sparsely distributed. Although the main response regions could still be detected, the continuity of extremely fine and discontinuous structures was not sufficiently preserved, as reflected by incomplete recovery of local connectivity and missed detection of small regions.

Taken together, the limitations of the proposed method are mainly concentrated in three extreme scenarios: samples with extremely low contrast and blurred boundaries between defects and background, complex scenes in which the background textures are highly similar to target textures and easily induce false responses, and tiny structural targets that are slender, discontinuous, and weakly salient. Although the synergy among TDM, RPR, and SCM substantially improved the overall segmentation capability for complex industrial defects, there remains room for further improvement in recovering local boundaries, ultra-fine structures, and weak-response regions under these challenging conditions. In future work, stronger boundary supervision, multi-scale detail enhancement strategies, and uncertainty-guided post-refinement methods may be introduced to further improve robustness and generalization in complex industrial scenarios.

## 5. Conclusions

In this work, TDRPFormer was proposed for pixel-level steel surface defect segmentation under complex background interference. The framework combines texture debiasing, prediction-conditioned prototype routing, and high-resolution structural modulation to reduce normal-texture confusion and improve the recovery of defect regions.

Experiments on ESDIs-SOD and NEU-DET showed that TDRPFormer achieved the best overall segmentation performance among the compared methods. The ablation results further supported the complementary contributions of the three components. However, the multi-stage refinement and prototype-routing process introduced higher inference latency than the lightweight one-pass models. Therefore, the current design prioritizes segmentation reliability and structural recovery over maximum throughput.

From a practical perspective, the proposed framework is relevant to automated steel-surface inspection scenarios in which normal texture interference, weak defect responses, and ambiguous boundaries can reduce the reliability of pixel-level predictions. By suppressing normal-texture responses while preserving low-contrast and slender defect structures, TDRPFormer may provide more stable defect masks for downstream localization, area measurement, and quality assessment. These characteristics are particularly useful in inspection tasks that require accurate defect delineation rather than image-level defect classification alone. Nevertheless, the present evaluation was conducted on two public steel surface defect datasets. Further validation under different production lines, imaging devices, material conditions, and defect distributions is still required before broader industrial deployment.

Despite these encouraging results, the analysis of the failure cases showed that the proposed method may still suffer from local boundary deviation, missed small regions, or insufficient structural recovery in extremely challenging scenarios, such as very low contrast, high similarity between background and target textures, and ultra-slender discontinuous structures. These findings indicate that complex industrial defect segmentation remains constrained by reliable boundary modeling, ultra-fine detail representation, and potential distribution shifts across practical inspection conditions. Future work will investigate stronger boundary constraints, multi-scale detail enhancement strategies, uncertainty-guided post-refinement, and broader cross-scenario validation to further improve robustness and generalization in complex industrial environments.

## Figures and Tables

**Table 1:** Quantitative comparison results on the ESDIs-SOD dataset.

Method	M↓	$mF_{\beta }$↑	$F_{\beta }^{w}$↑	$S_{\alpha }$↑	$mE_{\xi }$↑
UNet	0.0646	0.7208	0.6124	0.8086	0.8154
PSPNet	0.1048	0.5908	0.4347	0.7203	0.7093
DeepLabv3+	0.0752	0.6742	0.5578	0.7798	0.7802
UperNet	0.0924	0.6302	0.4956	0.7546	0.7376
SegFormer	0.0430	0.7554	0.7230	0.8502	0.8524
DefectSAM	0.0248	0.8486	0.8284	0.8907	0.9466
WPFormer	0.0196	0.8630	0.8660	0.8940	0.9480
DEPANet	0.0215	0.8510	0.8490	0.8830	0.9360
MINet	0.0286	0.8235	0.8209	0.8820	0.9182
TDRPFormer (Ours)	0.0185	0.8780	0.8824	0.9070	0.9626

↓ represents the lower the value, the better; ↑ represents the higher the value, the better.

**Table 2:** Quantitative comparison results on the NEU-DET dataset.

Method	M↓	$mF_{\beta }$↑	$F_{\beta }^{w}$↑	$S_{\alpha }$↑	$mE_{\xi }$↑
UNet	0.0357	0.8047	0.7851	0.8669	0.8973
PSPNet	0.0495	0.7284	0.6851	0.8325	0.8394
DeepLabv3+	0.0431	0.7708	0.7369	0.8502	0.8704
UPerNet	0.0455	0.7599	0.7185	0.8442	0.8634
SegFormer	0.0369	0.7952	0.7752	0.8652	0.8940
DefectSAM	0.0272	0.8607	0.8413	0.8924	0.9545
WPFormer	0.0219	0.8781	0.8813	0.8972	0.9615
DEPANet	0.0228	0.8745	0.8776	0.8924	0.9563
MINet	0.0319	0.8191	0.8127	0.8759	0.9174
TDRPFormer (Ours)	0.0210	0.8824	0.8858	0.9017	0.9662

↓ represents the lower the value, the better; ↑ represents the higher the value, the better.

**Table 3:** Category-conditioned evaluation of TDRPFormer on the NEU-DET test set. Groups are defined by the original labels present in the annotation files and are not mutually exclusive.

Method	No.Test Images	M↓	$mF_{\beta }$↑	$F_{\beta }^{w}$↑	$S_{\alpha }$↑	$mE_{\xi }$↑
Inclusion (In)	441	0.0207	0.8546	0.8588	0.8880	0.9620
Patches (Pa)	303	0.0286	0.9198	0.9199	0.9085	0.9666
Scratches (Sc)	421	0.0200	0.8771	0.8845	0.9060	0.9692
All test images	887	0.0210	0.8824	0.8858	0.9017	0.9662

↓ represents the lower the value, the better; ↑ represents the higher the value, the better.

**Table 4:** Quantitative comparison results on the SD-saliency-900 dataset under zero-shot cross-dataset evaluation.

Method	M↓	$mF_{\beta }$↑	$F_{\beta }^{w}$↑	$S_{\alpha }$↑	$mE_{\xi }$↑
UNet	0.1212	0.5969	0.4457	0.7118	0.7335
PSPNet	0.1601	0.4573	0.3458	0.6451	0.6348
DeepLabv3+	0.1041	0.5806	0.4566	0.7246	0.7274
UPerNet	0.1665	0.4863	0.3659	0.6581	0.6451
SegFormer	0.0585	0.6913	0.6425	0.8107	0.8126
DefectSAM	0.0328	0.8252	0.8191	0.8692	0.9267
WPFormer	0.0193	0.8471	0.8456	0.8901	0.9412
DEPANet	0.0248	0.8351	0.8269	0.8755	0.9317
MINet	0.0373	0.7596	0.7485	0.8384	0.8809
TDRPFormer (Ours)	0.0189	0.8500	0.8482	0.8920	0.9440

↓ represents the lower the value, the better; ↑ represents the higher the value, the better.

**Table 5:** Ablation results of different core module configurations on the ESDIs-SOD and NEU-DET datasets. Values are reported as mean ± standard deviation over three independent runs.

Method	TDM	RPR	SCM	ESDIs-SOD				NEU-DET			
				M↓	$F_{\beta }^{w}$↑	$S_{\alpha }$↑	$mE_{\xi }$↑	M↓	$F_{\beta }^{w}$↑	$S_{\alpha }$↑	$mE_{\xi }$↑
Baseline				0.0308 ± 0.0007	0.7812 ± 0.0048	0.8728 ± 0.0024	0.9254 ± 0.0019	0.0304 ± 0.0006	0.8217 ± 0.0042	0.8829 ± 0.0023	0.9443 ± 0.0017
Baseline + TDM	✓			0.0247 ± 0.0006	0.8316 ± 0.0039	0.8912 ± 0.0021	0.9461 ± 0.0017	0.0254 ± 0.0005	0.8511 ± 0.0036	0.8936 ± 0.0020	0.9564 ± 0.0016
Baseline + RPR		✓		0.0275 ± 0.0007	0.8127 ± 0.0041	0.8844 ± 0.0022	0.9387 ± 0.0018	0.0271 ± 0.0006	0.8424 ± 0.0038	0.8901 ± 0.0021	0.9527 ± 0.0016
Baseline + SCM			✓	0.0282 ± 0.0007	0.8069 ± 0.0043	0.8871 ± 0.0022	0.9402 ± 0.0017	0.0276 ± 0.0006	0.8389 ± 0.0039	0.8914 ± 0.0021	0.9538 ± 0.0016
Baseline + TDM + RPR	✓	✓		0.0214 ± 0.0005	0.8548 ± 0.0030	0.8996 ± 0.0018	0.9557 ± 0.0014	0.0228 ± 0.0005	0.8723 ± 0.0031	0.8981 ± 0.0018	0.9621 ± 0.0014
Baseline + TDM + SCM	✓		✓	0.0221 ± 0.0005	0.8495 ± 0.0032	0.9018 ± 0.0017	0.9573 ± 0.0013	0.0233 ± 0.0005	0.8692 ± 0.0032	0.8992 ± 0.0017	0.9628 ± 0.0013
Baseline + RPR + SCM		✓	✓	0.0243 ± 0.0006	0.8394 ± 0.0036	0.8967 ± 0.0019	0.9508 ± 0.0015	0.0246 ± 0.0006	0.8614 ± 0.0035	0.8968 ± 0.0019	0.9594 ± 0.0015
TDRPFormer (Ours)	✓	✓	✓	0.0185 ± 0.0004	0.8824 ± 0.0025	0.9070 ± 0.0015	0.9626 ± 0.0012	0.0210 ± 0.0004	0.8858 ± 0.0027	0.9017 ± 0.0014	0.9662 ± 0.0011

✓ indicates that the module has been added; otherwise, a regular convolution is used instead. ↓ represents the lower the value, the better; ↑ represents the higher the value, the better.

**Table 6:** Ablation results of different texture-debiasing and gating strategies. Values are reported as mean ± standard deviation over three independent runs.

Setting	ESDIs-SOD			NEU-DET		
	M↓	$F_{\beta }^{w}$↑	$S_{\alpha }$↑	M↓	$F_{\beta }^{w}$↑	$S_{\alpha }$↑
Identity	0.0243 ± 0.0007	0.8398 ± 0.0042	0.8965 ± 0.0023	0.0248 ± 0.0006	0.8573 ± 0.0039	0.8941 ± 0.0022
Direct-Add	0.0229 ± 0.0006	0.8477 ± 0.0038	0.8998 ± 0.0020	0.0238 ± 0.0006	0.8634 ± 0.0036	0.8966 ± 0.0019
Concat-Conv	0.0219 ± 0.0006	0.8538 ± 0.0035	0.9014 ± 0.0019	0.0230 ± 0.0005	0.8688 ± 0.0034	0.8983 ± 0.0018
Gate-Residual	0.0206 ± 0.0005	0.8672 ± 0.0029	0.9049 ± 0.0017	0.0220 ± 0.0005	0.8781 ± 0.0030	0.9001 ± 0.0016
TDM (Ours)	0.0185 ± 0.0004	0.8824 ± 0.0025	0.9070 ± 0.0015	0.0210 ± 0.0004	0.8858 ± 0.0027	0.9017 ± 0.0014

↓ represents the lower the value, the better; ↑ represents the higher the value, the better.

**Table 7:** Ablation results of confidence and boundary-response cues. Values are reported as mean ± standard deviation over three independent runs.

Setting	ESDIs-SOD			NEU-DET		
	M↓	$F_{\beta }^{w}$↑	$S_{\alpha }$↑	M↓	$F_{\beta }^{w}$↑	$S_{\alpha }$↑
w/o confidence cue	0.0206 ± 0.0005	0.8709 ± 0.0032	0.9041 ± 0.0018	0.0224 ± 0.0005	0.8763 ± 0.0031	0.9000 ± 0.0017
w/o boundary cue	0.0198 ± 0.0005	0.8767 ± 0.0029	0.9050 ± 0.0017	0.0217 ± 0.0004	0.8802 ± 0.0028	0.9007 ± 0.0016
w/o confidence and boundary cues	0.0222 ± 0.0006	0.8614 ± 0.0037	0.9012 ± 0.0020	0.0239 ± 0.0006	0.8686 ± 0.0036	0.8974 ± 0.0019
Full model	0.0185 ± 0.0004	0.8824 ± 0.0025	0.9070 ± 0.0015	0.0210 ± 0.0004	0.8858 ± 0.0027	0.9017 ± 0.0014

↓ represents the lower the value, the better; ↑ represents the higher the value, the better.

**Table 8:** Effect of different query numbers with the prototype number fixed at $N_{p}$ = 16. Values are reported as mean ± standard deviation over three independent runs.

N	ESDIs-SOD			NEU-DET		
	M↓	$F_{\beta }^{w}$↑	$S_{\alpha }$↑	M↓	$F_{\beta }^{w}$↑	$S_{\alpha }$↑
8	0.0199 ± 0.0005	0.8736 ± 0.0031	0.9055 ± 0.0018	0.0221 ± 0.0005	0.8807 ± 0.0030	0.9006 ± 0.0017
16	0.0185 ± 0.0004	0.8824 ± 0.0025	0.9070 ± 0.0015	0.0210 ± 0.0004	0.8858 ± 0.0027	0.9017 ± 0.0014
32	0.0188 ± 0.0004	0.8809 ± 0.0026	0.9068 ± 0.0016	0.0212 ± 0.0004	0.8849 ± 0.0026	0.9015 ± 0.0015
64	0.0191 ± 0.0005	0.8796 ± 0.0028	0.9061 ± 0.0017	0.0215 ± 0.0005	0.8836 ± 0.0028	0.9009 ± 0.0016

↓ represents the lower the value, the better; ↑ represents the higher the value, the better.

**Table 9:** Effect of different prototype numbers with the query number fixed at N = 16. Values are reported as mean ± standard deviation over three independent runs.

$N_{p}$	ESDIs-SOD			NEU-DET		
	M↓	$F_{\beta }^{w}$↑	$S_{\alpha }$↑	M↓	$F_{\beta }^{w}$↑	$S_{\alpha }$↑
4	0.0205 ± 0.0006	0.8686 ± 0.0034	0.9044 ± 0.0020	0.0229 ± 0.0006	0.8761 ± 0.0033	0.8998 ± 0.0019
8	0.0193 ± 0.0005	0.8787 ± 0.0029	0.9060 ± 0.0017	0.0217 ± 0.0005	0.8822 ± 0.0028	0.9010 ± 0.0016
16	0.0185 ± 0.0004	0.8824 ± 0.0025	0.9070 ± 0.0015	0.0210 ± 0.0004	0.8858 ± 0.0027	0.9017 ± 0.0014
32	0.0188 ± 0.0004	0.8806 ± 0.0026	0.9066 ± 0.0016	0.0213 ± 0.0004	0.8844 ± 0.0026	0.9013 ± 0.0015

↓ represents the lower the value, the better; ↑ represents the higher the value, the better.

**Table 10:** Effects of different interaction and modulation forms in RPR and SCM. Values are reported as mean ± standard deviation over three independent runs.

Module	Setting	ESDIs-SOD			NEU-DET		
		M↓	$F_{\beta }^{w}$↑	$S_{\alpha }$↑	M↓	$F_{\beta }^{w}$↑	$S_{\alpha }$↑
RPR	Context only	0.0227 ± 0.0006	0.8581 ± 0.0033	0.8991 ± 0.0019	0.0240 ± 0.0006	0.8684 ± 0.0032	0.8973 ± 0.0018
RPR	Prototype only	0.0234 ± 0.0006	0.8519 ± 0.0036	0.8974 ± 0.0020	0.0245 ± 0.0006	0.8649 ± 0.0035	0.8958 ± 0.0019
RPR	Context + Prototype	0.0185 ± 0.0004	0.8824 ± 0.0025	0.9070 ± 0.0015	0.0210 ± 0.0004	0.8858 ± 0.0027	0.9017 ± 0.0014
SCM	Identity	0.0217 ± 0.0005	0.8566 ± 0.0034	0.8987 ± 0.0019	0.0231 ± 0.0005	0.8705 ± 0.0032	0.8979 ± 0.0018
SCM	Concat-Conv	0.0204 ± 0.0005	0.8689 ± 0.0030	0.9032 ± 0.0017	0.0222 ± 0.0005	0.8780 ± 0.0029	0.9003 ± 0.0016
SCM	Residual Modulation (Ours)	0.0185 ± 0.0004	0.8824 ± 0.0025	0.9070 ± 0.0015	0.0210 ± 0.0004	0.8858 ± 0.0027	0.9017 ± 0.0014

↓ represents the lower the value, the better; ↑ represents the higher the value, the better.

**Table 11:** Quantitative consistency analysis of the deterministic confidence cue on the complete ESDIs-SOD test set. The statistics were computed from the stage-3 prediction prior and TDM background weights.

Analysis Category	Quantity	Value
Prediction reliability	Mean confidence of correct pixels	0.9925
	Mean confidence of incorrect pixels	0.8124
	Mean normalized entropy of correct pixels	0.0075
	Mean normalized entropy of incorrect pixels	0.1876
Confidence stratification	Pixel error rate, C<0.50	0.4754
	Pixel error rate, 0.50≤C<0.75	0.4441
	Pixel error rate, 0.75≤C<0.90	0.4054
	Pixel error rate, C≥0.90	0.0220
TDM background selection	Background-weight mass within GT background	0.9851
	GT background-area fraction	0.8848
	Background-weight purity enrichment	1.1133

**Table 12:** Comparison of model complexity and inference efficiency. Params are reported in millions (M), FLOPs in billions of floating-point operations (G), Latency as the forward inference time per image, and FPS as the number of processed frames per second. Except for DefectSAM, all methods were evaluated at an input resolution of 384 × 384. Because DefectSAM uses a default input resolution of 1024 × 1024, its complexity and speed are not directly comparable on a strictly identical basis, although they still reflect its overall computational burden.

Method	Input	Params(M)	FLOPs(G)	Latency(ms)	FPS
UNet	384 × 384	29.06	114.16	6.79	147.21
PSPNet	384 × 384	48.96	100.50	11.72	85.36
DeepLabv3+	384 × 384	43.58	99.27	13.82	72.34
UPerNet	384 × 384	66.40	133.31	15.03	66.52
SegFormer	384 × 384	3.72	4.18	13.34	74.94
DefectSAM	1024 × 1024	93.74	488.24	71.18	14.05
TDRPFormer	384 × 384	26.74	15.88	56.47	17.71

## Data Availability

The data underlying the results presented in this paper are not publicly available at this time but may be obtained from the authors upon reasonable request.
